# ﻿Discovering the diversity of Acarosporaceae (Acarosporales, Lecanoromycetes) with carbonized epihymenial accretions in North America

**DOI:** 10.3897/mycokeys.122.162675

**Published:** 2025-09-11

**Authors:** Kerry Knudsen, Jana Kocourková, Eva Kondrysová, Tereza Pušová, Jason Hollinger, Steve Leavitt, John McCarthy, Lucie Jedličková, Martin Westberg

**Affiliations:** 1 Czech University of Life Sciences Prague, Faculty of Environmental Sciences, Department of Ecology, Kamýcká 129, Praha – Suchdol, 165 00, Czech Republic Czech University of Life Sciences Prague Praha - Suchdol Czech Republic; 2 Herbarium, Department of Biology, Western Carolina University, Cullowhee, NC 28723, USA Western Carolina University Cullowhee United States of America; 3 Department of Biology & M.L. Bean Life Science Museum, Brigham Young University, Provo, UT 84602, USA Brigham Young University Provo United States of America; 4 Herbarium, Department of Biology, Laurentian University, Sudbury, Ontario, P3E 2C6, Canada Laurentian University Sudbury Canada; 5 Museum of Evolution, Uppsala University, Norbyvägen 16, Uppsala, Sweden Uppsala University Uppsala Sweden

**Keywords:** Beta-tubulin, integrative taxonomy, keys, new combination, species diversity, undescribed taxa

## Abstract

Six new species are described: *Acarospora anthracina*, *A. austrooccidentalis*, *A. oscurensis*, and *A. profusa* from southwestern North America; *A. aquatica* from wetlands in eastern North America; and *A. minuta* from the Holarctic flora of Canada. In total, 13 species of Acarosporaceae with carbonized epihymenial accretions are known from North America. No European taxa of *Acarospora* or *Sarcogyne* with carbonized epihymenial accretions were found in North America, except *A. lapponica*, which is part of the Holarctic flora in Alaska, and the lichenicolous calciphyte *Sarcogyne pusilla*. A key to North American Acarosporaceae with carbonized epihymenial accretions is provided. *Sarcogyne joshuaensis* is transferred to *Acarospora*.

## ﻿Introduction

Vězda (1978) erected the genus *Polysporina* in the Acarosporaceae (Acarosporales, Lecanoromycetes). It was a morphological genus concept that encompassed all species of *Sarcogyne* with black lecideine apothecia with epihymenial carbonized accretions of umbos and/or gyrose structures.

The key characteristic for identifying *Polysporina* taxa was the carbonized epihymenial accretions. Species were then separated by substrate (calcareous vs. non-calcareous rock or soil). Various commonly used diagnostic characters were employed to distinguish species, for instance, ascospore size, chemistry, epilithic vs. endolithic thallus, hymenium height, immersion in substrate, paraphyses width, parasitism, and the reaction of hymenial gel to IKI (Kantvilas 1998; Knudsen and Kocourková 2008; Knudsen et al. 2016, 2021). *Polysporina* eventually contained 17 species (Index Fungorum 2024).

Westberg et al. (2015) clearly demonstrated with a four-gene phylogenetic study that lecideine apothecia with carbonized epihymenial accretions had evolved independently several times in the Acarosporaceae and that *Polysporina* was polyphyletic. The type of the genus, *Polysporina simplex* nom. illegit. (=*Acarospora privigna*), was recovered in the *Acarospora* clade, with the rest of the taxa divided between the *Acarospora* and *Sarcogyne* clades. Only with phylogenetic analysis – not morphology and anatomy – can individual species be placed in either the *Acarospora* or *Sarcogyne* clades. By 2023, the genus *Polysporina* was no longer accepted on authoritative checklists in Europe and North America (Esslinger 2021; Nimis 2021; Printzen et al. 2022; Roux et al. 2022; Malíček et al. 2023).

Our objective is the taxonomic, phylogenetic, and phylogenomic study of the rich species diversity of Acarosporaceae in North America, where 127 described species occur and new species continue to be discovered (Knudsen et al. 2023, 2025). In this paper, we continue our study of Acarosporaceae with carbonized epihymenial accretions in North America (Knudsen 2007; Knudsen and Kocourková 2008; Knudsen et al. 2011, 2016, 2021, 2022).

## ﻿Material and methods

### ﻿Herbarium study

We studied collections in ASU, BRY-C, COLO, and NY, as well as in the private herbaria of Jana Kocourková and Kerry Knudsen (hb. K&K) and Jason Hollinger and Nastassja Noell (hb. H&N). We examined specimens of *A. minuta* collected by John McCarthy and eventually divided between CANL and various herbaria. The morphology of specimens was examined with dissecting microscopes. The anatomy of hand sections was examined and measured in water at 1000× with compound microscopes. The amyloid reaction of the hymenial gel and subhymenium was tested with fresh, undiluted IKI (Merck’s Lugol for the gram staining method, Sigma-Aldrich 1.09261) (see protocol in Knudsen et al. 2024). The ascus stain was studied in IKI (Hafellner 1993). Thin-layer chromatography (TLC) in solvents A and C was performed to identify secondary metabolites (Orange et al. 2001). The endolithic thallus was studied by scratching the substrate surface of specimens and examining it with a dissecting microscope. Upon completion of the study, holotypes and some isotypes and paratypes were deposited in BRY-C, CANL, NBM, NF, SBBG, and PRM.

### ﻿Imaging

Macrophotographs were taken with a digital camera (Olympus DP74) mounted on an Olympus SZX 16 stereomicroscope, using PROMICRA QuickPHOTO INDUSTRIAL 4 software, and stacked with the Olympus DeepFocus 3.5 module to increase depth of field. Microphotographs were taken with an Olympus DP74 digital camera mounted on an Olympus BX51 light microscope fitted with Nomarski interference contrast, using PROMICRA QuickPHOTO INDUSTRIAL 4 software. The figure plates were processed with the Figure Maker module fitted to the same software.

### ﻿DNA extraction, PCR amplification, and sequencing

DNA was extracted from 44 dried herbarium specimens with lecideine apothecia and carbonized epihymenial accretions (Suppl. material 1). Genomic DNA was extracted from lichenized thalli via the Invisorb Spin Plant Mini Kit, according to the manufacturer’s protocol with slight modifications (i.e., DNA was diluted to 50 μL instead of the 100 μL recommended by the kit) and incubated in buffer for 15 minutes before final centrifugation. Total extracted DNA was stored at −20 °C. The quality and yield of isolated DNA were checked on a 1% agarose gel, and DNA concentration and purity were measured precisely using a UVS‐99 spectrophotometer (ACTGene). The selected genes for this study were the nuclear ribosomal internal transcribed spacer (nrITS; White et al. 1990), the large subunit of the nuclear ribosomal DNA (nLSU; Vilgalys and Hester 1990), the small subunit of the mitochondrial ribosomal DNA (mtSSU; Zoller et al. 1999), and the coding sequence of the β-tubulin gene (β-TUB; Einax and Voigt 2003). The ITS, nLSU, β-TUB, and mtSSU regions were amplified via polymerase chain reaction (PCR). Each reaction contained 1 μL (20–25 ng) of extracted genomic DNA, 10 μL of 2× MyTaq Red DNA Polymerase (Bioline), 8.2 μL of water, and 0.4 μM of forward/reverse primer (10 μM) for a total reaction volume of 20 μL. Conditions for ITS and mtSSU rDNA: initial denaturation at 95 °C for 5 min, followed by five cycles (95 °C for 33 s, 56 °C for 30 s, and 72 °C for 30 s), then ten cycles (95 °C for 30 s, 54 °C for 30 s, and 72 °C for 30 s), and twenty cycles (95 °C for 30 s, 50 °C for 30 s, and 72 °C for 30 s), with a final extension at 72 °C for 15 min. For the nLSU: initial denaturation at 95 °C for 1 min, followed by five cycles (95 °C for 30 s, 55 °C for 30 s, and 72 °C for 60 s), and finally 30 cycles (95 °C for 30 s, 52 °C for 30 s, and 72 °C for 60 s), with a final extension at 72 °C for 10 min. For β-TUB: initial denaturation at 95 °C for 1 min, followed by five cycles (95 °C for 30 s, 60 °C for 30 s, and 72 °C for 60 s), and finally 30 cycles (95 °C for 15 s, 55 °C for 30 s, and 72 °C for 45 s), with a final extension at 72 °C for 10 min. Before sequencing, the PCR products were purified using the enzymatic method ExoSap-IT Express Reagent (Thermo Fisher Scientific, Inc.), according to the manufacturer’s protocol. PCR products were run on a 1.0% agarose gel via electrophoresis and stained with ethidium bromide for 20 min. Purified PCR products, water, and forward primer (8 μL total volume) were sent to BIOCEV, Vestec, Czech Republic. Sequences were checked against the UNITE and NCBI databases for contamination. All newly generated sequences were deposited in GenBank (Suppl. material 1).

### ﻿Sequence alignment and phylogenetic analysis

We produced new sequences from 44 samples, and additional sequences were obtained from GenBank (Suppl. material 1). We analyzed a combined matrix of the four genes, which included a total of 153 sequences. The sequences were proofread and concatenated manually into a single dataset using SEQUENCHER version 5.4.6. Sequences were aligned using the multiple sequence alignment online service MAFFT version 7.110 with the “G-INS-I” strategy (Katoh and Toh 2008; http://mafft.cbrc.jp/alignment/server/). Indels longer than 1 bp were coded using the simple gap coding method (Simmons and Ochoterena 2000) as implemented in SEQSTATE 1.4.1 (Müller 2005). A partition homogeneity test (incongruence length difference) was performed in PAUP* version 4.0a169 (Swofford 2002) under 1,000 replicates, using a heuristic search to assess congruence among the ITS, nLSU, β-TUB, and mtSSU sequence partitions. with heuristic search was performed under one thousand replicates between the ITS, nLSU, β-TUB, and mtSSU sequences by PAUP* version 4.0a169 (Swofford 2002) to determine whether the partitions were homogeneous for the test of congruence. The combined matrix included a total of 2,738 characters of aligned DNA sequences from mitochondrial and nuclear genes: ITS rDNA (447 bp), nLSU rDNA (886 bp), mtSSU rDNA (709 bp), and β-tubulin (696 bp). Of these characters, 1,683 were constant, 234 were variable and parsimony-uninformative, and 821 were parsimony-informative. For phylogenetic analyses, the GTR+I model was selected as the best-fitting model of nucleotide substitution for each gene, based on the Akaike Information Criterion using JMODELTEST 2.1.10 (Darriba et al. 2012). A phylogenetic tree was constructed using MRBAYES 3.2.2 (Ronquist and Huelsenbeck 2003). Input data were formatted for MRBAYES via the FABOX online converter tool (http://birc.au.dk/~palle/php/fabox/fasta2mrbayes.php; Villesen 2007). Sequences of *Pycnora sorophora* were included as an outgroup. Three replicate analyses with four chains each were run for 30,000,000 generations, sampling every 1,000^th^ generation. After this number of generations, the average standard deviation of split frequencies reached a value lower than 0.01, indicating convergence. The MCMC analysis of the four concatenated genes was run for 30,000,000 generations. The data were also analyzed using maximum likelihood (ML) methods (Suppl. material 2). Tree searches for ML analyses were executed under the GTR+GAMMA nucleotide substitution model in RAxML v.8.2.10 (Stamatakis 2014). The Bayesian phylogenetic tree with posterior probabilities and the ML phylogenetic tree with 1,000 pseudoreplicates and a nonparametric bootstrap approach were visualized using FigTree v.1.4.0 and rooted with *Pycnora sorophora* (Rambaut 2012) The ML tree is available in Suppl. material 2. The final alignment is accessible in the TreeBASE database (https://treebase.org/) under submission ID 31614.

## ﻿Results and discussion

The sequences from Westberg et al. (2015) are included in our phylogeny. All specimens in our phylogeny with the genus name *Polysporina* are from Europe (mainly Fennoscandia). The only exception is *Polysporina subfuscescens*LN810847, which is a parasitic taxon from the Mojave Desert in Joshua Tree National Park in southern California, with 90% sequence similarity to *Acarospora destructans*. We have not sequenced any species with carbonized epihymenial accretions from Joshua Tree National Park, except two type specimens of *A. destructans* (Knudsen et al. 2022). None of these European *Polysporina* taxa were resolved taxonomically in Westberg et al. (2015), which was only a study of the evolution of taxa with carbonized epihymenial accretions.

**Figure 1.**
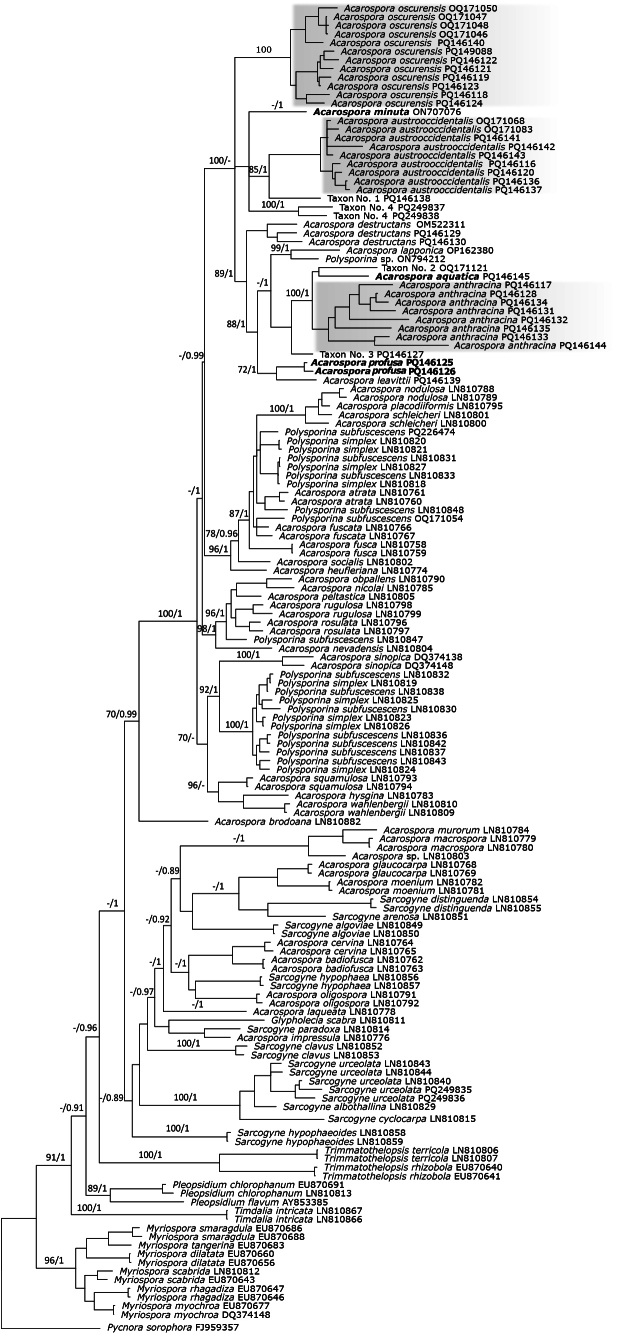
Bayesian inference tree obtained by phylogenetic analysis using a combined dataset of ITS, mtSSU, nLSU, and β-TUB sequences of 153 members of Acarosporaceae. Maximum likelihood bootstrap values (ML ≥ 70%) and Bayesian posterior probability (BPP ≥ 0.95) are indicated above branches (ML/BPP). *Pycnora sorophora* was used as an outgroup.

In a well-supported North American clade in *Acarospora* (Fig. 1), we recovered, using integrative taxonomy, six new species for science, which we describe in this paper: *Acarospora anthracina*, *A. aquatica*, *A. austrooccidentalis*, *A. minuta*, *A. oscurensis*, and *A. profusa* (shaded or in bold in Fig. 1). We recovered four additional taxa (*Acarospora* taxon No. 1–4). *Acarospora* taxon No. 1 is sister to *A. austrooccidentalis*. It was collected in Wayne County, Utah, an area rich in Acarosporaceae diversity, and is anatomically indistinguishable from *A. austrooccidentalis* except for having a well-developed mycelial base, and is genetically distinct. *Acarospora* taxon No. 2 is a collection from California of *A. simillima* nom. prov. It is the only analog to *A. privigna* we have found so far in North America and will be treated in a future study when more sequences are published. *Acarospora* taxon No. 3 is most closely related to *A. anthracina* but does not occur on calcareous rock and does not have euamyloid hymenial gel. *Acarospora* taxon No. 4 is a high-elevation montane taxon on siliceous rock from the La Sal Range in Manti-La Sal National Forest in Utah (Leavitt 18541) and the Sawtooth Range in Idaho (Hollinger 17524). It is probably widespread in the Rockies. The Utah specimen has no ascospores.

One cannot rule out that No. 1 and No. 3 might represent taxa produced from introgression or hybridization, a subject we are studying in the *Acarospora strigata* group. As proposed in Vences et al. (2024), substantially divergent but admixing phylogeographical lineages can conveniently be named as subspecies, thus avoiding taxonomic oversplitting and inflation. This is a possible classification for taxa No. 1 and No. 3. Taxa No. 2 and No. 4 represent distinct species in need of more specimens before they can be described. We recognize 13 described species in North America with carbonized epihymenial accretions, including the six species described in this paper.

Two species not endemic to North America were recovered in the well-supported clade of North American taxa: the Holarctic species *Acarospora lapponica*, reported from Alaska by Magnusson (1929), and a sister taxon, *Polysporina* sp. ON794212, from Spain, collected by M. Westberg. As discussed in Knudsen et al. (2025), the name *A. lapponica* may include several taxa and requires molecular sampling throughout its Holarctic range.

*Acarospora brodoana*, described and only known from the San Bernardino Mountains in southern California (Knudsen et al. 2016), was recovered at the base of the *Acarospora* clade in an isolated position, as in Westberg et al. (2015). In our experimental trees, *A. brodoana* is most closely related to the terricolous lichen *A. thelococcoides*, endemic to a range stretching from central California to Baja California in Mexico.

In the *Sarcogyne* clade, we recovered *Sarcogyne cyclocarpa* and *S. urceolata* from Europe and *S. albothallina* from North America. The occurrence of *S. cyclocarpa* or *S. urceolata* in North America is unverified, though both could be members of the Holarctic flora of northern North America. Specimens that have been determined as *S. urceolata* in southwestern North America (Knudsen 2007) are recovered as *A. anthracina* (see further discussion under that species).

Specimens identified as *S. cyclocarpa* from North America were recovered as *A. aquatica* (Knudsen et al. 2011). *Sarcogyne albothallina* is the only North American *Sarcogyne* with carbonized epihymenial accretions (Knudsen et al. 2016).

Interestingly, North American *Acarospora anthracina* in an earlier experimental tree with only three genes, without β-tubulin, was recovered in the *Sarcogyne* clade with *S. cyclocarpa* and *S. urceolata*, but in the final tree with added β-tubulin, it was recovered in the *Acarospora* clade.

### ﻿Taxonomy

#### ﻿ Acarospora anthracina

Taxon classificationFungiAcarosporalesAcarosporaceae

K.Knudsen, Kocourk. & Kondrysová sp. nov.

AED3C353-E524-5D4E-8AFD-D28B382216A2

857356

Fig. 2

##### Type.

U.S.A., Nevada, Lincoln Co., Mormon Mountains, N-facing cliffs WSW of Horse Springs, ca. 250 m E of peak x7189, base of massive N-facing limestone cliffs, pinyon-juniper zone, 36.937, -114.457, alt. 2100 m, on limestone, 10 Aug. 2019, J. Hollinger 23119 (holotype, Bry-L-0050241), J. Hollinger 23110 (topotype, BRY-L-0050234).

**Figure 2.**
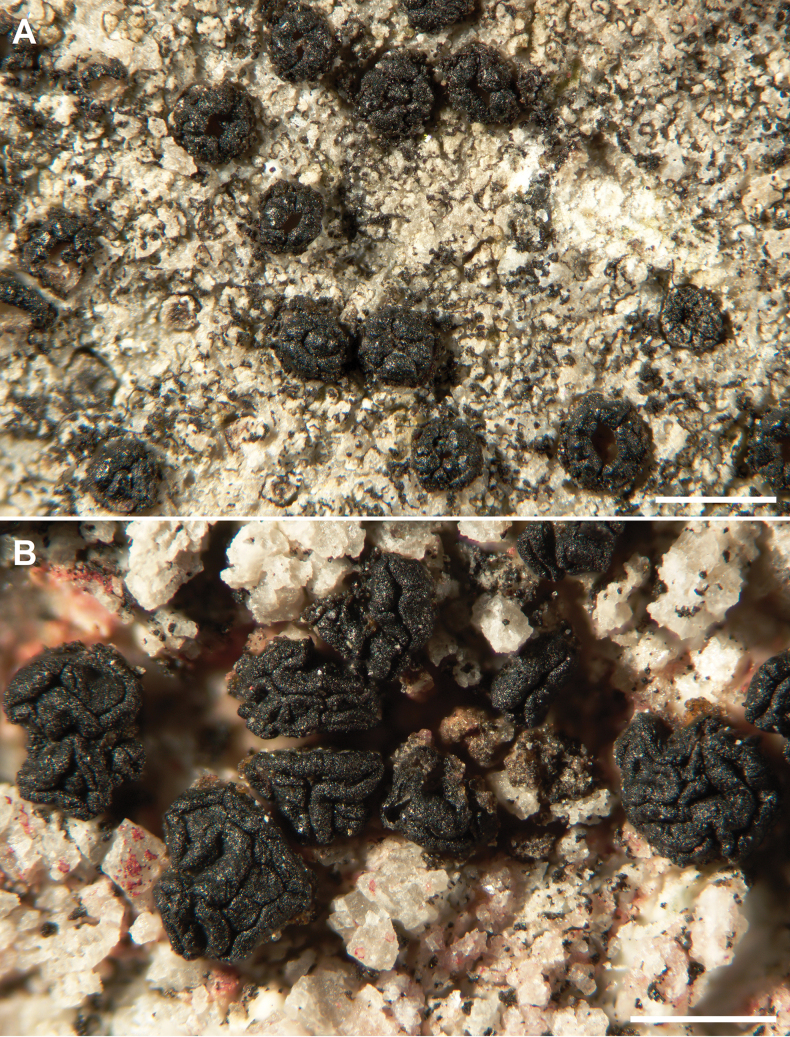
*Acarospora anthracina*, Hollinger 23119 Holotype (A), Topotype (B). A. Habit of immersed thallus with young black apothecia; B. Fully developed gyrose apothecial disc. Scale bars: 500 µm (A, B).

##### Diagnosis.

Similar to the calciphyte *Sarcogyne urceolata* in Europe and Africa, which is also lichenicolous. but with different sequence data and recovered in the *Acarospora* clade rather than in the *Sarcogyne* clade.

##### Etymology.

Its name refers to its black apothecia.

##### Description.

Thallus endolithic of hyphae mostly 2 µm wide, algae usually at base of apothecia in substrate, algal cells 5–10 µm wide in loose clusters, sometimes absent, especially beneath the smallest apothecia. Apothecia black, broadly attached, 0.1–0.4(–0.5) mm wide, rarely larger than 1.0 mm and replicating by division, 0.1–0.3 mm thick, smallest on hardest limestone. Margin entire in young apothecia, becoming knobby or uneven and/or segmented, irregular when apothecia replicating by division, in largest specimens ca. 100 µm wide, outer layer ca. 80 µm wide, carbonized, inner layer hyaline, hyphae 1–2 µm wide, sometimes 40 µm wide in smallest specimens. Disc black, rarely pruinose, without carbonized epihymenial accretions when young, usually forming one umbo, usually higher than margin, sometimes with gyrose structures. Hymenium (60–)80–100 µm tall, epihymenium black. Paraphyses 1–2 µm wide, apices barely expanded in black caps, hymenial gel IKI+ dark blue, euamyloid. Asci 40–70 × 10–25 µm, ascospores variable 2.0–5.5 × 1.0–2.5 µm, sometimes globose 2×2 µm mixed with ellipsoid ascospores (n = 40), sometimes with an oil drop. Subhymenium 10–30 µm tall, IKI+ blue. Hypothecium continuous with margin and endolithic thallus, hyphae mostly 2 µm thick. Pycnidia not observed. Not producing secondary metabolites.

##### Habitat and distribution.

On limestone and other calcareous rock in Arizona, California (White Mountains), Nevada, and Utah. Expected to have wider distribution in North America.

##### Additional specimens examined.

U.S.A., • Arizona, Mohave Co., Grand Canyon Parashant National Monument, 36.67, -113.6311, alt. 1500 m, on limestone, 20 May 2003, K. G. Sweats 206 (ASU); • California, Mono Co., White Mountains, Patriarch Grove, Bristlecone Pine Forest, 37.5275, -118.1980, alt. 3453 m, on dolomite rock chips, 17 Jul. 2002, S. Tucker 38026 (SBBG); • Colorado, Rio Blanco Co, BLM land southwest of Rangely, off CR23, Piceance Basin, rocky steppe dominated by junipers, 39.9739, -108.8641, 1874 m, on calcareous sandstone, 18 Apr. 2025, E. Manzitto-Tripp 11423 (COLO); • Nevada, Lincoln Co., Golden Gate Range, Continental Pass, 37.953, -115.399, alt. 1650 m, on limestone, J. Hollinger 11031 & N. Noell (BRY-L-0049261); • Highland Peak, north slope near top of western peak, massive limestone cliffs surrounded by *Abies concolor*-*Pinus longaeva* forest high on steep north slope, 37.899, -114.586, alt. 2750 m, on ledge in limestone slot, 2 Jun 2016, J. Hollinger 12539, 12544 & N. Noell (hb. H&N); • Highland Peak, ridge to west of western peak, high limestone ridge with old *Cercocarpus ledifolius* forest, 37.897, -114.587, alt. 2835 m, on vertical limestone cliff, 2 Jun 2016, J. Hollinger 12651, 12656, 12669 & N. Noell (hb. H&N); • Mount Irish, high on N ridge with some large limestone outcrops and open conifer forest with *Abies concolor*, *Pinus longaeva*, *P. monophylla*, *Juniperus osteosperma*, *J. scopulorum* and *Artemisia nova*, 37.646, -115.401, alt. 2600 m, on limestone talus, 14 Jun 2016, J. Hollinger 13181 & N. Noell (hb. H&N), (BRY- L-0050241); • Pershing Co., Humboldt Range, Black Canyon, 40.5482, -118.1887, alt. 2565 m, on limestone, 10 Oct. 2014, J. Hollinger 8156, 8157 (hb. H&N); • Utah, Garfield Co., Aquarius Plateau, near head of Sweetwater Creek slopes of conifer woodland, 37.827 -111.899. alt. 2650 m, on calcareous sandstone, 5 May 2017, J. Hollinger 17278, 17279 (hb. H&N); • Grand Co., La Sal Range, Monti La Sal National Forest on Barrow Ridge, 38.5008, -109.2287, alt. 3560 m, on calciferous sandstone, 21 Aug. 2018, S. Leavitt 18629a (BRY-L-0051913); • Kane Co., Fifty Mile Bench, pinyon juniper woodland on N to NE-facing slope, 37.3015 -111.1035, alt. 1930 m, on calcareous sandstone, 9 May 2017, J. Hollinger 17397 (hb. H&N); • Millard Co., Pavant Mountains, side valley above Chalk Creek Canyon, 38.9263, -112.2117, alt. 1994 m, outcrops of rocks, on limestone, 10 Sept. 2024, J. Kocourková 11551 & K. Knudsen (hb. K&K); • Chokecherry Creek, Fillmore Canyon Rd., 38.9186, -112.2026, alt. 2283 m, outcrops of rocks above creek valley, on limestone, 11 Sept. 2024, J. Kocourková 11553 & K. Knudsen (hb. K&K); • San Juan Co., La Sal Range, Dark Canyon, 38.4444, -109.2321. alt. 3500 m, on calcareous sandstone, 20. Aug. 2018. S. Leavitt 18541 (BRY-L-0051395); • Washington Co., Big Mountain, 37.513, -113.655, alt. 2200 m, on calcareous sandstone, 21 Sept. 2015, J. Hollinger 19709 & N. Noell (BRY-L-0052022).

##### Notes.

*Sarcogyne anthracina* differs from *S. urceolata* in usually having a higher hymenium (60–)80–100 vs. 60–80 µm, in not being lichenicolous, and in having different sequence data placing it in the *Acarospora* clade and not in the *Sarcogyne* clade (Thor et al. 2023).

There are many North American specimens in the Consortium of Lichen Herbaria identified as *Sarcogyne urceolata*, but this name was used for any specimen on limestone (CLN 2024). We do not consider *S. urceolata* as verified in North America. It could occur in the Holarctic flora of northern North America. At the same time, *A. anthracina* may occur throughout North America.

#### ﻿ Acarospora aquatica

Taxon classificationFungiAcarosporalesAcarosporaceae

K.Knudsen, Kocourk. & Kondrysová sp. nov.

91DA771F-EFF2-555F-8B80-ED16FE271093

857358

Fig. 3

##### Type.

U.S.A. • New York. Rockland Co., Harriman State Park, between Conklin Mountain and Wanaksink Lake, vicinity of Tuxedo Rock, 41.1883, -74.1247, alt. 366 m, on quartz crystals on granite dome between *Sphagnum* depressions, 9 March 2008, J. C. Lendemer 11525 (NY-holotype).

**Figure 3.**
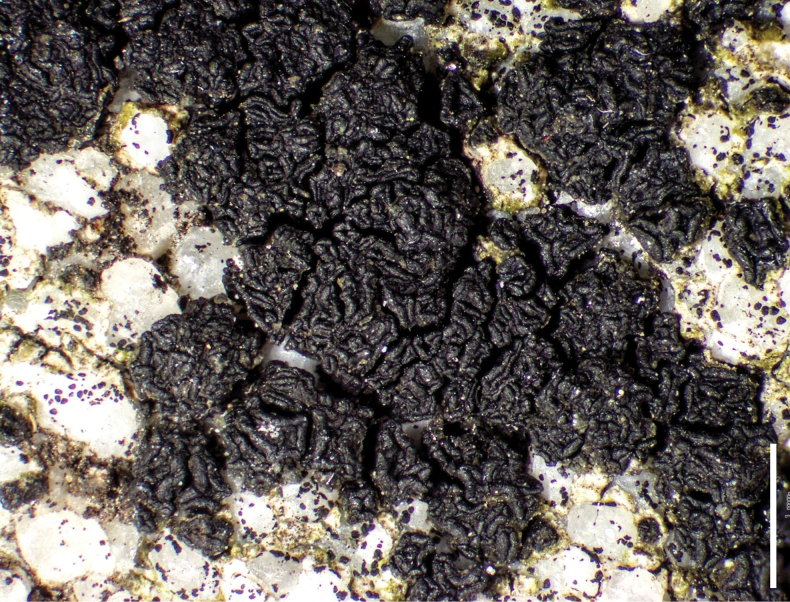
*Acarospora aquatica* Lendemer 11525. Habit of the thallus with compound apothecia with gyrose disc. Scale bar: 1 mm.

##### Diagnosis.

Differing from *Sarcogyne cyclocarpa* in having a wetlands ecology and forming compound apothecial structures by replication through division.

##### Etymology.

Named for its occurrence in swamps and wetlands, an unusual character in the family.

##### Description.

Thallus endolithic, algal layer usually visible at base of apothecia, algal cells 5–10 µm wide, often forming continuous layer below compound apothecia. Apothecia dispersed, becoming compound, ca. 0.2–0.4 mm wide, 0.2–0.3 mm thick, including the stipe, lower surface carbonized. Margin ca. 50 μm wide, outer layer carbonized, inner layer hyaline to light brownish color, widths variable, sometimes gobs of melanin build-up on the outer surface. Disc black with umbos and/or gyrose structures. Hymenium 100–125 µm tall, paraphyses 1–2 µm wide, apices barely expanded, hymenial gel IKI+ blue to red, hemiamyloid. Asci 60–80 × 10–20 µm, ascospores 4–5 × 2 μm. Subhymenium 40–80 µm tall, IKI+ blue. Hypothecium usually indistinct, continuous with endolithic thallus and margin of solitary apothecia or outer wall of compound apothecia. Pycnidia not observed. Not producing secondary metabolites.

##### Habitat and distribution.

On granite or quartz in granite, limestone, and Altamaha grit sandstone in wetlands in Connecticut, Georgia, New York, and West Virginia.

##### Additional specimens examined.

U.S.A. • Connecticut, Windham Co., Eastford, Natchaug State Park, Cat Den Swamp, red maple swamp, along Fayette Road, 41.8431, -72.0547, no alt., on granite, 19 Sept. 2009, R.C. Harris 55703 (NY); • Georgia, Coffee Co., Broxton. Broxton Rocks TNC Preserve, High Point Outcrop. loblolly pine, water and blackjack oaks, 31.7422, -82.8536, no alt., on Altamaha grit sandstone; 10 Jul. 2007, E. Lay s.n. (NY); • New York, Jefferson Co., Three Mile Creek Barrens, hardwood swamp forest, 44.13, -76.1525. no alt, on adjacent alvar limestone pavements, 25 May 1997, R.C. Harris 40852 (NY); • West Virgina, Tuckerco Co., Blackwater Falls State Park, open fields and wetlands 39.1158, -79.4808, alt. 945 m, on rock, 23 Apr. 2001, R.C. Harris 44925 (NY).

##### Notes.

The species is probably occasionally submerged.

We first studied all specimens at NYBG in 2011 and published them as *Polysporina cyclocarpa* (Knudsen et al. 2011). We did limited dissection of specimens in 2023 and 2024 at BYU to preserve specimens. We base ascospore data on our previous study (Knudsen et al. 2011). All the specimens were old, and sequences were only recovered from the holotype.

#### ﻿ Acarospora austrooccidentalis

Taxon classificationFungiAcarosporalesAcarosporaceae

K. Knudsen, Kocourk. & Kondrysová sp. nov.

FC881413-6452-599E-9E6C-C11842A0DCA5

857359

Fig. 4

##### Type.

U.S.A. • New Mexico, Lincoln Co., Tularosa Basin, Oscura, Road 54, 33.4863, -106.0928, alt. 1475 m, SW-NE crest above the valley, southernmost hill, on soft acid sandstone, 14 March 2022, J. Kocourková 10842 (PRM-holotype, Hb. K&K-isotype).

**Figure 4.**
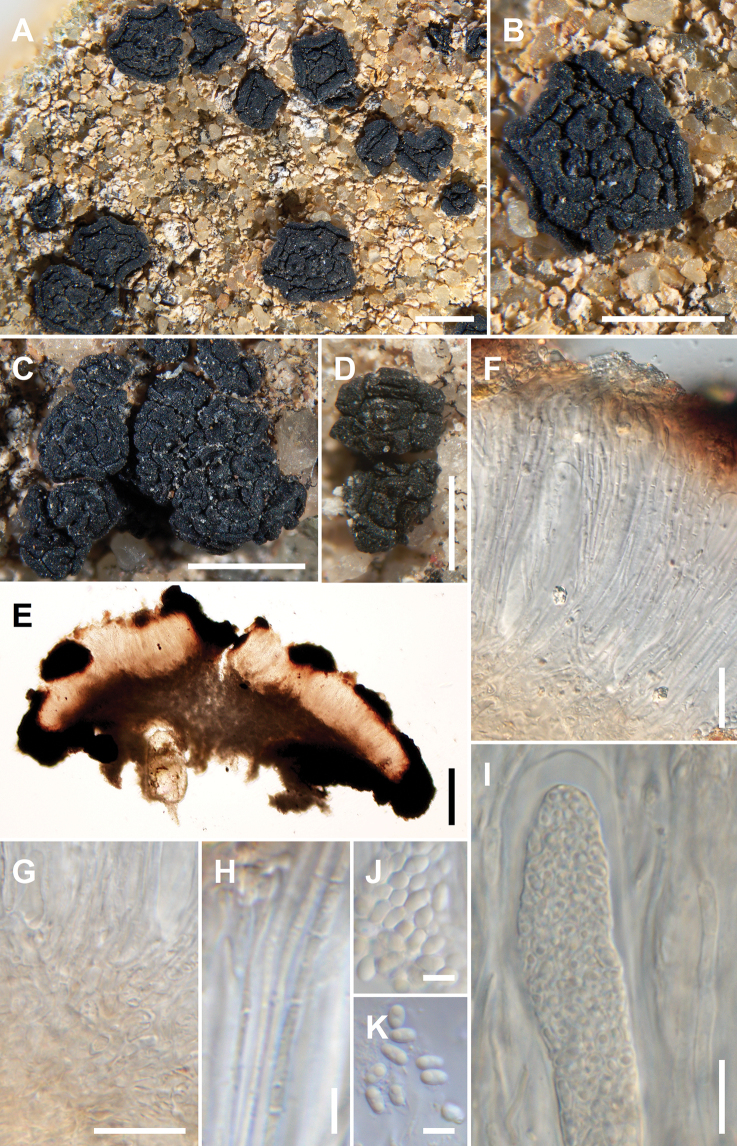
*Acarospora austrooccidentalis* Kocourková 11902.3 (A–C, E, I), Kocourková Holotype (F–H), Kocourková Isotype (D, J, K). A. Habit of the white ecorticate chasmolithic thallus with apothecia; B. Apothecium with gyrose disc; C. Apothecia forming clusters; D. Young apothecia with segmented margin; E. Apothecia compound with two hymenia; F. Hymenium of asci and paraphyses; G. Hypothecium; H. Non anastomosed very occasionally branched paraphyses; I. Ascus forming ascospores; J, K. Ascospores. Scale bars: 1000 µm (A–C); 500 µm (D); 200 µm (E); 20 µm (F, G); 10 µm (I); 5 µm (H, J, K).

##### Diagnosis.

Similar to *A. oscurensis* but differs especially in not producing initially apothecia with a smooth margin and without epihymenial accretions and in not forming white stromata with pycnidia, in having ellipsoid ascospores (1.5–)4.0–4.6(–5.1) × (1.0–)2.0(–2.8) µm vs. globose to broadly ellipsoid ascospores (1–)2–3(–6) × 1.0–3.4 µm, and in having thinner paraphyses 1.0–1.5(–2.8) µm vs. (1.5–)2–2.5(–3.5) µm.

##### Etymology.

Named for its southwestern distribution in North America.

##### Description.

Endolithic base or white ecorticate chasmolithic thallus in large-grained sandstone or with small white mycelial base, algal cells, 5–10 µm wide, scattered deep in substrate or sometimes below apothecia, sometimes forming a continuous algal layer between apothecia. Apothecia 0.5–1.6 mm wide, 0.3–0.4 mm tall, convex or not, sometimes compound with two hymenia, dispersed or occasionally forming clusters of apothecia with a mycelial base through replication by division. Margin segmented, usually in long linear sections at joints, 90–120 µm wide, outer layer carbonized, inner area hyaline, width variable, margin sometimes excluded. Disc black, usually gyrose with dense carbonized epihymenial accretions, often higher than margin. Hymenium (100–)120–150 µm tall, paraphyses 1.0–1.5(–2.5) µm wide, simple or slightly branched, non-anastomosed, apices unexpanded, hymenial gel IKI+ dark blue, euamyloid, or turning pale blue and fading to pale green, or blue turning red in squash, hemiamyloid, not of diagnostic value. Asci variable from 100 × 10–40 to 40 × 15 µm in same hymenium, ellipsoid ascospores (1.5–)4.0–4.6(–5.1) × (1.0–)2.0(–2.8) µm (n = 40). Subhymenium 40–50 µm tall, IKI+ dark blue, euamyloid. Hypothecium 20–100 µm tall, hyphae 1–2 µm wide or gelatinized and filled with substrate crystals, continuous with margin and endolithic thallus. No pycnidia observed. Not producing secondary metabolites.

##### Habitat and distribution.

Currently known from California, Nevada, New Mexico, and Utah, on non-calcareous sandstone, occasionally calciferous sandstone. One specimen was collected on cracked granite. Several specimens were collected on calciferous volcanic tuff. It can occur with *A. oscurensis*, as it does at its type locality in New Mexico.

##### Additional specimens examined.

U.S.A. • Arizona, Yavapai Co., Coconino National Forest, Red Rock Ranger District, Transept Trail, southwest-facing cliff, 34.7907, -111.7894, alt. 1298 m, no date, G. Neil 609a (ASU); • California, Tuolumne Co., edge of Emigrant Wilderness, Huckelberry Trail through Kennedy Meadow, white pine, incense cedar, 38.2916, -119.333, alt. 1950–2025 m, on a cracked granite rock, 14 Aug. 1989, B. Ryan 24540-b (ASU); • Nevada, Lincoln Co. Antelope Canyon, 37.637, -114.535, alt.1500 m, on vertical quartzite outcrop, 30 Mar. 2016, J. Hollinger 11033 & N. Noell (BRY-L-0049263); • Clover Mountain, Tepee Rocks, base of loose ash-tuff cliff, 37.5958, -114.4407, alt. 1690 m, on ash-tuff, 31 May 2018, J. Hollinger 21365 (hb. H&N); • Jumbled Hills, at base of east-facing ash-tuff cliff, 37.3070, -115.6142, alt. 1514 m, on calciferous ash-tuff, 28 May 2018, N. Noell 3909, 3917 & J. Hollinger, J. Hollinger 21350 & N. Noel (hb. H&N); • New Mexico, Lincoln Co., Tularosa Basin, Oscura, Road 54, 33.4863, -106.0928, alt. 1475 m, SW-NE crest above the valley, southernmost hill, on soft acid sandstone, 14 March 2022, J. Kocourková 10902, 11003 (hb. K&K); • Utah, Emery Co., San Rafael Desert, vicinity of Three Canyons overlook, on sandstone on rim west of the Green River, 38.7087, -110.1245, alt. 1320 m, 15 Apr. 2023, S. Leavitt et al. 23067, 23092 (BRY-C); • Kane Co., Glen Canyon Recreational Area, in Navajo sandstone canyon, 37.311, -110.977, alt. 1160 m, on sandstone, 17 May 2019, S. Leavitt s.n. (BRY-L-0050518); • San Juan Co., Bears Ears National Monument, between the UT 211 Rd. and sandstone crest, 37.9836, -109.5003, alt. 1869 m, thin juniper and shrubby pine vegetation on gently sloping terrain, on sandstone rocks, 22 Sept. 2024, J. Kocourková 11559 & K. Knudsen (hb. K&K); • La Sal Mountains, La Sal Loop Rd, top of small SW oriented crest, 38.4532, -109.3750, alt. 1866 m, on HCl–sandstone, 21 Sept. 2024, J. Kocourková 11554 & K. Knudsen (hb. K&K); • Wayne Co., Capital Reef National Park, Cassidy Arch Trail, 38.2637, -111.2159, no alt., on Navajo sandstone, 12 June 1992, Larry St. Clair 14030 (BRY-L-0030684); • ca. 2 miles from entrance to Maze District of Canyonlands National Park, 38.2212, -110.2130, alt. 1948 m, on exposed sandstone bedrock, 9 April 2014, J. Hollinger 6321 & N. Noell (hb. H&N) • Highway 95, on sandstone, 3 Oct., 1986, J. W. Thomson (ASU).

##### Notes.

High hymenium, thin paraphyses, and ellipsoid ascospores distinguish *Acarospora austrooccidentalis*. *Acarospora oscurensis* also has a high hymenium but differs in having globose to broadly ellipsoid ascospores and thicker paraphyses. Unfortunately, the ascospore size of the two species can overlap, with *A. oscurensis* sometimes having some ascospores that are not broadly ellipsoid but ellipsoid. Paraphyses width can also be a difficult character, as occasional specimens of both species can have a predominance of paraphyses 2 µm wide. Lichens did not evolve for lichenologists. *Acarospora oscurensis* also differs in having young apothecia with a smooth margin and a disc without carbonized epihymenial accretions as well as occasionally white stromata.

The specimens from the Jumbled Hills in Lincoln County, Nevada, on eroding calcareous tuff had the lowest hymenia at 100 µm high and formed a chasmolithic thallus from substrate erosion and were not as robust as specimens on large-grained HCl–sandstone (Noell 3909, 3917).

#### ﻿ Acarospora minuta

Taxon classificationFungiAcarosporalesAcarosporaceae

K. Knudsen, J. W. McCarthy & Kondrysová sp. nov.

2ECDB4AF-8449-529E-8D76-CB699ECA62E2

857360

Fig. 5

##### Type.

Canada • Newfoundland and Labrador, Newfoundland, Western Newfoundland, Port-au-Port East, Route 462, Pine Tree Road to the top of Table Mountain (south of Point-au-Mal), alpine coastal limestone barren, 48.5953, -58.6611, alt. 345 m, on siliceous rock among limestone scree, 3 Aug. 2017, J. W. McCarthy 3347 & C. McCarthy (CANL-holotype).

**Figure 5.**
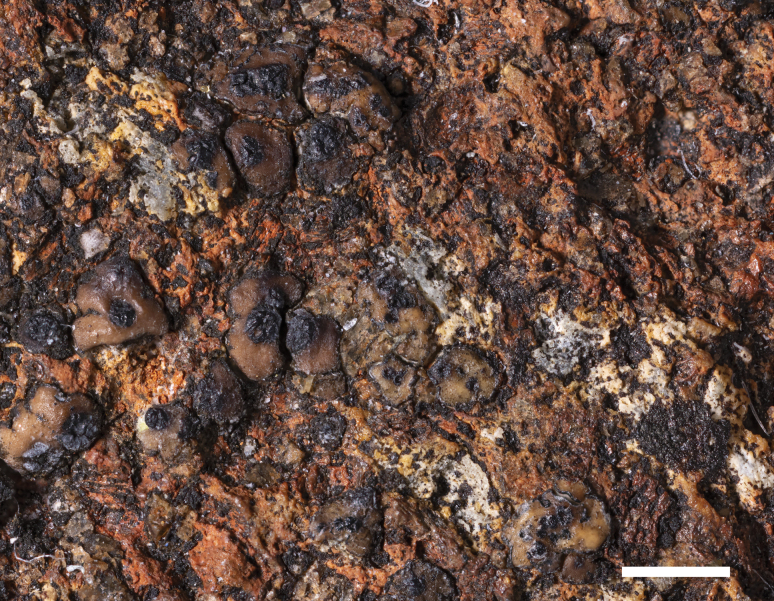
*Acarospora minuta* McCarthy 3347 Holotype. Thallus of dispersed thick brown areoles, one apothecium per areole. Scale bar: 2 mm.

##### Diagnosis.

Similar to *Polysporina limborinella* in having small ascospores but differs in having an epilithic brown thallus and occurring in Canada and not in Switzerland (Nimis et al. 2018).

##### Etymology.

Named for the small size of its ascospores.

##### Description.

Hypothallus endosubstratal, no algae observed. Thallus of dispersed thick brown areoles, beginning at base of apothecia and expanding to 1 mm wide and 300–400 μm thick. Upper surface shiny brown, epruinose. Epicortex up to 15 μm thick and continuous. Cortex 20–50 μm thick, narrow upper layer brown, less than 10 μm thick, lower layer hyaline, cells irregular in shape, 2–4 μm wide. Algal layer 80–100 μm thick, uninterrupted, algal cells 10–15 μm wide, continuous or not beneath apothecia. Medulla obscure, sometimes with a few scattered algal cells, hyphae 2–4 μm wide. Apothecia usually one per areole, occasionally two to six, 0.2–0.4 mm wide, 100–200 μm tall. Margin elevated up to 50 μm above the disc, dividing into four or five segments, thin, hyphae 1–2 μm wide, outer layer black 10 μm wide, inner layer hyaline. Disc black, with or without one umbo. Hymenium 90–140 μm tall, paraphyses 1 μm wide, apices unexpanded, some branching, often filled with oil drops, hymenial gel IKI+ red or blue turning red in squash, hemiamyloid. Asci 45–60 × 15–20 µm, ascospores mostly 2–3 × 0.5–1.0 μm (n = 40), thin ellipsoid, sometimes oil drops, several hundred per ascus. Subhymenium 20–50 μm tall, IKI+ blue. Hypothecium continuous with margin and thallus, sometimes distinct directly below apothecia, hyaline and narrow, sometimes golden yellow. No pycnidia observed. Not producing secondary metabolites.

##### Habitat and distribution.

Known only from two localities, growing on siliceous rocks among limestone on Table Mountain in western Newfoundland, Canada, and on mafic gabbro rock in Annieopsquotch Mountains in the southwestern interior of Newfoundland, Canada.

##### Additional specimens examined.

Canada • Newfoundland and Labrador, Newfoundland, Port-au-Port East, Route 462, Pine Tree Road to the top of Table Mountain (south of Point-au-Mal), limestone scree slopes along forested gully stream, alpine coastal limestone barren, 48.601, -58.6542, alt. 250 m, on siliceous rock among limestone scree, 3 Aug. 2017, J. W. McCarthy 3346 & C. McCarthy (NBM); • Port-au-Port East, Route 462, Pine Tree Road to the top of Table Mountain (south of Point-au-Mal), alpine, coastal limestone barren, 48.5953, -58.6611, alt. 345 m, on siliceous rock among limestone scree, 3 Aug. 2017, J. W. McCarthy 3348 & C. McCarthy (NFLD); • Port-au-Port East, Route 462, Pine Tree Road to the top of Table Mountain (south of Point-au-Mal), alpine, coastal limestone barren, 48.5953, -58.6611, alt. 345 m, on siliceous rock among limestone scree, 3 Aug. 2017, J. W. McCarthy 3349 & C. McCarthy (SBBG); • southwest Newfoundland, Burgeo Highway (Route 480), Annieopsquotch Mountains, 48.2804, -57.7051, alt. 535 m, on mafic rock, 9 Oct. 2018, J. W. McCarthy 3751 & C. McCarthy (CANL).

##### Note.

Sequences were only recovered from one of several specimens we studied.

Two other species with carbonized epihymenial accretions have epilithic thalli, *Sarcogyne albothallina* in our key and *Acarospora tasmaniensis*, occurring in soil crusts in Tasmania. Other taxa may rarely have a chasmolithic or epilithic thallus, usually formed by exposure of endolithic thallus by erosion of substrate.

#### ﻿ Acarospora oscurensis

Taxon classificationFungiAcarosporalesAcarosporaceae

K. Knudsen, Kocour. & Kondrysová sp. nov.

2EB21C0F-F665-5CD8-8A5D-1F9BA3D0227F

857361

Fig. 6

##### Type.

U.S.A. • New Mexico, Lincoln Co., Tularosa Basin, Oscura, Road 54, 33.4863, -106.0928, alt. 1475 m, SW-NE crest above the valley, southernmost hill, on soft acid sandstone, 14. Mar. 2022, K. Knudsen 19438 & J. Kocourková (SBBG-holotype).

**Figure 6.**
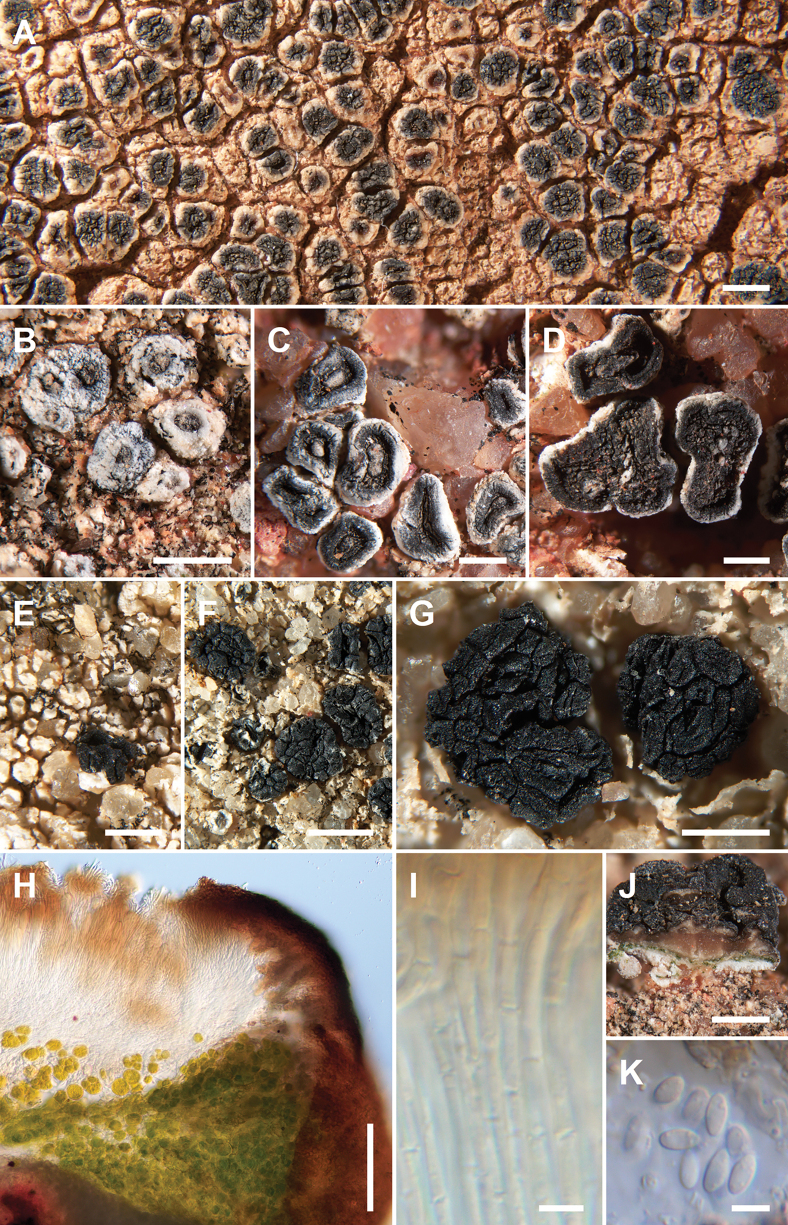
*Acarospora oscurensis*, Hollinger 6327 (A), Knudsen 19438 Holotype (B, H–J), Kocourková 10795.1 (C), Kocourková 10795.3 (D), Kocourková 10846 (E), Kocourková 11512 (F, G, K). A. Habit of the thallus with apothecia emerging from white stromata; B. White stromata with pycnidia; C. Elevated apothecia developing from pycnidia, with thick margin and umbo developing on the disc; D. Maturing elevated apothecia; E, F. White stromata and marginate apothecia developing gyrose surface; G. Fully developed immarginate gyrose apothecia; H. Vertical section through apothecium showing hyaline hymenium and algal layer; I. Septate and anastomosed paraphyses; J. Vertical section through apothecium under dissecting microscope; K. Spores. Scale bars: 1 mm (A, F); 500 µm (B–E, G, J); 100 µm (H); 5 µm (I, K).

##### Diagnosis.

Similar to *Acarospora austrooccidentalis* with a high hymenium, differing in having globose to broadly ellipsoid ascospores (1–)2–3(–6) × 1.0–3.4 µm vs. ellipsoid ascospores (1.5–)4.0–4.6(–5.1) × (1.0–)2.0(–2.8), thicker paraphyses (1.5–)2.0–2.5(–3.5) µm vs. 1.0–1.5(–2.0) µm, in occasionally producing white stromata, and with young apothecia often having in early development a smooth margin and lacking epihymenial accretions.

##### Etymology.

Named for Oscura in the Tularosa Basin of the Chihuahuan Desert in New Mexico, where we first collected this species.

##### Description.

Thallus endolithic up to 0.2 mm deep, up to 5 cm wide, algal cells ca. 5–10 µm wide, algal layer variable, continuous below and between apothecia, the sandstone often spongy when wet to the touch, or discontinuous deep in substrate. Apothecia round to irregular, 0.3–1.3 mm wide, 0.4–0.5 mm tall, sometimes elevated on white mycelial base or emerging from white stromata. Margin thick, 100–250 µm wide, outer layer carbonized, inner layer reddish to hyaline, widths variable, hyphae 1–2 µm wide, rim smooth when young, slightly elevated above the disc, becoming segmented. Disc reddish-brown to black, at first usually without epihymenial accretions, then developing an umbo or several small dots of melanin, eventually dense and sometimes gyrose, excluding the margin. Hymenium (100–)120–150(–180) µm tall, epihymenium uneven (10–)20–30 µm tall, paraphyses (1.5–)2.0–2.5(–3.5) µm wide, branched and anastomosed, apices expanded to 4.5 µm with black pigment mark, hymenial gel IKI+ light blue, turning red in squash, hemiamyloid. Asci 120–160 × 10–30 µm, often cylindrical, rarely becoming inflated, ascospores usually broadly ellipsoid, sometimes with small globose ascospores (1–)2–3(–6) × 1.0–3.4 µm, variable (n = 40), oil drops absent or one large oil drop, sometimes a second smaller one. Subhymenium up to 30 µm tall, IKI+ blue. Hypothecium 20–30 µm tall, with hyphae 1–2 µm thick, continuous with margin. Occasionally with white stromata 100–300 µm wide, a black dot often on top and some scattered algae cells in a thalline wall around pycnidia. Pycnidia up to 200 µm across with long, thick conidiogenous cells 30 × 3 µm, producing abundant conidia 2–3 × 1 µm. Eventually producing a single apothecium from each stroma. Not producing secondary metabolites.

##### Habitat and distribution.

On acid and calciferous sandstone and volcanic ash in Arizona, Colorado, Nevada, New Mexico, and Utah.

##### Additional specimens examined.

U.S.A. • Arizona, Maricopa Co., Sierra Estrella Regional Park, Corgett Wash, 33.2833, -112.3333, alt. 850 m, 20 Oct.1974, T. H. Nash III (ASU); • Colorado, Garfield Co., Flat Tops Wilderness, forested area just above Crater Lake, 39.8166, -107.4166, alt. 3140 m, on siliceous rocks, 24 June 1992, T. H. Nash III 31921(ASU); • Nevada, Lincoln. Co., Rainbow Canyon, large volcanic ash formation, 37.4959, -114.5827, alt. 1240 m, on sunny rhyolite ash, 13 Aug. 2019, Hollinger 23226a (BRY-L-0052277); • New Mexico, Lincoln Co., Tularosa Basin, Oscura, 33.4863, -106.0928, alt. 1475 m, SW-NE crest above the valley, southernmost hill, on soft acid sandstone, 14 March 2022, J. Kocourková 10795 (3 specimens, hb. K&K, SBBG); • 14 March 2022, J. Kocourková 10797 (2 specimens, hb. K&K, BYU), 10852 (2 specimens, hb. K&K, PRM); • Utah, Emery Co., 39.0945 -110.7392, alt. 1621 m, in the “Little Grand Canyon” of San Rafael River, south side of San Rafael River near sandstone butte above river on hard sandstone, 28 April 2023, S. Leavitt et al. 23124, 23126, 23129 (BRY-C); • 38.6839 -110.1695, alt. 1405 m, 15 April 2023, S. Leavitt et al. 23028, 23034 (BRY-C); • San Rafael Desert, vicinity of Three Canyons overlook, on sandstone on rim west of the Green River, 38.7087, -110.1245, alt. 1320, 15 Apr. 2023, S. Leavitt et al. 23077 (BRY-C); • San Juan Co., Bears Ears National Monument, between the UT 211 Rd. and sandstone crest, 37.9836, -109.5003, alt. 1869 m, thin juniper and shrubby pine vegetation on gently sloping area, on sandstone rocks, 22 Sept. 2024, J. Kocourková 11560 & K. Knudsen (hb. K&K), Knudsen 19532 & J. Kocourková (SBBG); • La Sal Mts., La Sal Loop Rd, top of small SW oriented crest, alt. 1866 m, 38.4532, -109.3750, on HCl–sandstone, 21 Sept. 2024, J. Kocourková 11512, 11556 & K. Knudsen (hb. K&K); • Wayne Co., Capital Reef National Park, Cassidy Arch Trail, 38.2637, -111.2159, no alt., on Navajo sandstone, 12 June 1992, Larry St. Clair 14030 (BRY-L-0030690).

##### Notes.

The high hymenium and broadly ellipsoid ascospores are characteristic of *Acarospora oscurensis*, as well as the tendency to have thicker paraphyses than *A. austrooccidentalis* (see also discussion under the latter species).

*Acarospora oscurensis*, like *Sarcogyne similis* and *S. poeltii*, produces stromata that contain pycnidia but which eventually produce apothecia (Knudsen et al. 2011, picture of stromata of *S. similis* as the synonym *A. reebiae*). It differs from these two species in the stromata being white and not black. As with *S. similis* and *S. poeltii*, apothecia may also emerge directly from the endolithic thallus or form by replication by division. So far white stromata are rare in collections examined but common at the type locality. They usually occur when the thallus is continuous between apothecia.

The apothecia of *Acarospora oscurensis* often do not have a continuous endolithic algal layer between apothecia and are elevated by a white mycelial base (which is not a remnant of stromata) and can have a distinct algal layer directly beneath the hypothecium.

*Acarospora oscurensis* can be easily determined by its young apothecia initially having a smooth margin and lacking epihymenial accretions on non-calcareous sandstone. But in emergent apothecia in sandstone with a porous top layer of large grains, the apothecia may have already developed an umbo and/or segmenting margin before they are fully emergent.

*Acarospora oscurensis* can be misdetermined as *A. leavittii*, which can sometimes have all small, broadly ellipsoid ascospores that are not fully developed to 10 × 7 µm, which are released at any size during good habitat conditions for lichenization. But *A. leavittii* generally will have longer ascospores (3.5–)4.0–5.0–7.0(–10.0) × (2.0–)2.5–3.0(–5.0) µm vs. (1–)2–3(–4) × 1–3 µm and a deeper subhymenium up to 60 µm vs. up to 30 µm tall (Knudsen et al. 2022).

#### ﻿ Acarospora profusa

Taxon classificationFungiAcarosporalesAcarosporaceae

K. Knudsen, Kocourk. & Kondrysová sp. nov.

6E35FACA-A19D-512E-AFD4-FDC2C894E147

857362

Fig. 7

##### Type.

U.S.A. • Utah, Garfield Co., Box Death Hollow Wilderness Area, east of Pine Creek Road, on Middle Jurassic - Late Jurassic HCl-sandstone escarpments above Pine Creek, 37.862, -111,631, alt. 1940 m, 20 May 2023, S. Leavitt 23203 (BRY-C-holotype), S. Leavitt 23187 (BRY-C, topotype).

**Figure 7.**
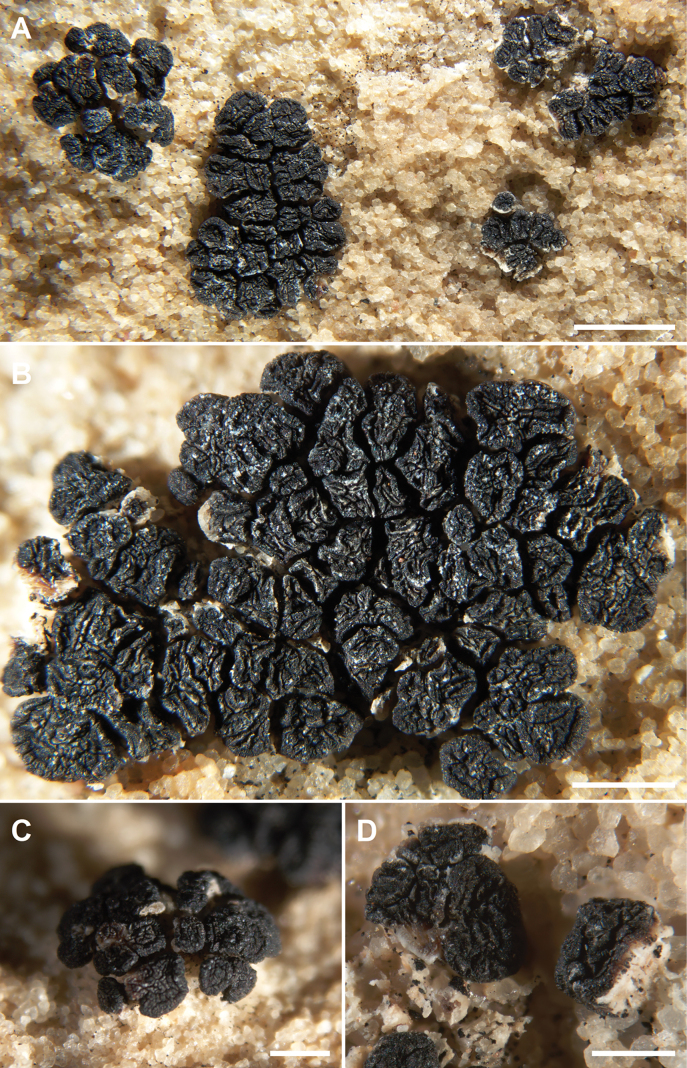
*Acarospora profusa*, Leavitt 23203 Holotype. A. Habit of the immersed thallus with fascicles of apothecia; B. Fascicle with more than 50 apothecia; C. Young stipitate fascicle of apothecia; D. Apothecium with white stipe of mycelial bundles. Scale bars: 2 mm (A); 1 mm (B); 500 µm (C, D).

##### Diagnosis.

Similar to *Sarcogyne aquatica* forming structures of compound apothecia but differing in forming aggregates of subdividing apothecia on a widening mycelial base, forming elevated fascicles on the end of interconnected “stipes” of vertical hyphae up to 0.3 mm high, continuing to replicate by division, and with up to 70 interconnected apothecia.

##### Etymology.

Named for the profusion of thin ascospores and of interconnected apothecia.

##### Description.

Thallus endolithic, algal layer below the mycelial base of apothecia, algal cells mostly 5–7 µm wide. Apothecia 0.2–1.0 mm wide. Margin with or without distinct segmented margins, round to irregular, varying in width, 30–40(–100 µm), outer layer black, one cell to 30–50 µm wide, inner layer hyaline, sometimes excluded. Disc black, gyrose, no umbos, epihymenial accretions 30–40 µm tall. Apothecia solitary at first but quickly replicating by division, forming elevated fascicles up to 0.3 mm high of (2–)10–70 apothecia. Eventually the mycelial base replicates by division, separating one aggregate of apothecia from another aggregate of apothecia. The mycelial base is filled with crystals and formed by vertical hyphae mostly 1 µm wide becoming bundles of intertwined hyphae ca. 5 µm wide, forming “stipes” elevating apothecia. Hymenium (80–)100–120 µm tall, paraphyses 1–2 µm wide, apices in black gel caps, hymenial gel IKI+ blue or red (if blue, turning red in squash), hemiamyloid. Asci thin, cylindrical to inflated, 100–90 × 10–20 µm, hundreds of ascospores, 1.0 × 1.0–1.5 µm, thin ellipsoid, sometimes with oil drop. Subhymenium indistinct, to 30 µm tall, IKI+ blue. Hypothecium indistinct. No pycnidia observed. Apothecia and/or epilithic thallus. producing low amounts of norstictic acid, spot test either negative or K+ faint yellow mist, not producing crystals, but positive in solvents A and C.

##### Habitat and distribution.

On non-calcareous sandstone in Utah.

##### Notes.

*Acarospora profusa* is easily determined by the thin ascospores and the fascicles of up to 70 elevated apothecia on a mycelial base. The elevating “stipes” of apothecia are formed by the vertical hyphae of the mycelial base growing upward to elevate apothecia. Eventually the mycelial base splits, forming “two islands” of fasciculate apothecia.

Though *Acarospora leavittii* often has dispersed apothecia, it can sometimes form similar-looking aggregates of apothecia but differs from *A. profusa* in not being elevated and instead being directly attached to a communal mycelial base that is dividing. *Acarospora leavittii* also differs in not having thin ellipsoid ascospores but globose to broadly ellipsoid ascospores that can eventually become as large as 7 × 10 µm.

## ﻿Conclusion

From a limited sampling of North American specimens with carbonized epihymenial accretions, we have recovered six species new to science, for a total of 13 described species with carbonized epihymenial accretions occurring in North America. Four undescribed *Acarospora* taxa with carbonized epihymenial accretions were recovered (*Acarospora* taxa No. 1–4) and require further study. A wider sampling from North America may recover more new species for science and may discover more described species from Europe than *Acarospora lapponica* and *Sarcogyne pusilla*.

With the description of seven more Acarosporaceae from North America – six in this paper and *Acarospora joshuaensis* from California – we recognize, in our study area of North America north of Mexico, 134 described species (Knudsen et al. 2025; Knudsen and Kocourková 2025; Suppl. material 3). In southwestern North America, north of Mexico, a center of diversity for the family, 108 described species occur, representing 80.60% of the total for North America north of Mexico. In California, with the report of *Acarospora austrooccidentalis* and the description of *A. joshuaensis*, the number of Acarosporaceae reported from the state rises to 64 species (Knudsen et al. 2025), the most for a single state.

### ﻿Key to Acarosporaceae with carbonized epihymenial accretions in North America

**Table:** 

1	In wetlands	***Acarospora aquatica* (this paper)**
–	Not in wetlands	**2**
2	Thallus epilithic	**3**
–	Thallus endolithic [rarely chasmolithic (visible mixed with substrate granules) or exposed by erosion]	**4**
3	Thallus brown, ascospores narrow, 1 µm wide, not producing traces of 4-O-methylhiascic Acid	***Acarospora minuta* (this paper)**
–	Thallus white, ascospores wider than 1 µm wide, producing traces of 4-O-methylhiascic acid	***Sarcogyne albothallina* (Knudsen et al. 2016)**
4	On calcareous rock, lichenicolous or not	**5**
–	On non-calcareous rock, sometimes also calciferous sandstone	**6**
5	Not lichenicolous	***Acarospora anthracina* (this paper)**
–	Lichenicolous on various lichens but also non-parasitic when hosts absent	***Sarcogyne pusilla* (Knudsen and Kocourková 2008, Thor et al. 2023)**
6	Lichenicolous	**7**
–	Not lichenicolous	**8**
7	Pathogenic, on many hosts, sequences variable, ascospores ellipsoid	***Acarospora destructans* (Knudsen et al. 2022)**
–	Not pathogenic; on *Candelariella*, ascospores globose	***Acarospora lendermeri* (Knudsen and Kocourková 2020)**
8	Hypothecium black	***Acarospora brodoana* (Knudsen et al. 2016)**
–	Hypothecium not black	**9**
9	Apothecia in black stromata, sometimes with algae between outer wall and parathecium, Holarctic (Alaska)	***Acarospora lapponica* (Knudsen and Kocourková 2008)**
–	Apothecia not in black stromata, algae not present	**10**
10	Apothecia in fascicles of up to 70 apothecia elevated by “stipes” growing upward from mycelial base	***Acarospora profusa* (this paper)**
–	Apothecia not in fascicles, up to 70 apothecia elevated by “stipes,” growing upward from mycelial base	**11**
11	Ascospores (3.5–)4.0–5.0(–7.0–10) × (2.0–)2.5–3.0(–5.0) µm	***Acarospora leavittii* (Knudsen et al. 2021)**
–	Ascospores not longer than 4–6 µm and not wider 2–3.4 µm	**12**
12	Ascospores ellipsoid, usually (1.5–)4.0–4.6(–5.1) × (1.0–)2.0(–2.8) µm, without white stromata, young apothecia with epihymenial accretions, paraphyses mostly thin, 1.0–1.5(–2.0) µm wide	.***Acarospora austrooccidentalis* (this paper)**
–	Ascospores globose to broadly ellipsoid ascospores (1–)2–3(–6) × 1.0–3.4 µm, rarely with white stromata, young apothecia usually without epihymenial accretions, paraphyses not thin, mostly 2.0–3.4 µm wide	***Acarospora oscurensis* (this paper)**

## Supplementary Material

XML Treatment for Acarospora anthracina

XML Treatment for Acarospora aquatica

XML Treatment for Acarospora austrooccidentalis

XML Treatment for Acarospora minuta

XML Treatment for Acarospora oscurensis

XML Treatment for Acarospora profusa

## ﻿Additional information

### Use of AI

No use of AI was reported.

### Author ORCIDs

Kerry Knudsen https://orcid.org/0000-0001-5419-5729

Jana Kocourková https://orcid.org/0000-0001-5511-9752

Eva Kondrysová https://orcid.org/0000-0002-8337-3253

Tereza Pušová https://orcid.org/0009-0001-2566-5266

Jason Hollinger https://orcid.org/0000-0003-2465-2487

Steve Leavitt https://orcid.org/0000-0002-5034-9724

John McCarthy https://orcid.org/0009-0003-1531-300X

Lucie Jedličková https://orcid.org/0000-0002-1811-9224

Martin Westberg https://orcid.org/0000-0002-8346-0322

### Data availability

All of the data that support the findings of this study are available in the main text or Supplementary Information.

## Supplementary materials

Supplementary material 1 Sampled specimens included in the molecular phylogeny Data type pdf https://binary.pensoft.net/file/1413676This dataset is made available under the Open Database License (http://opendatacommons.org/licenses/odbl/1.0/). The Open Database License (ODbL) is a license agreement intended to allow users to freely share, modify, and use this Dataset while maintaining this same freedom for others, provided that the original source and author(s) are credited.Kerry Knudsen, Jana Kocourková, Eva Kondrysová, Tereza Pušová, Jason Hollinger, Steve Leavitt, John McCarthy, Lucie Jedličková, Martin Westberg

Supplementary material 2 Maximum likelihood tree obtained by phylogenetic analysis using a combined data set of ITS, mtSSU, nLSU, and β-TUB sequences of 153 members of Acarosporaceae Data type pdf Explanation note Maximum likelihood bootstrap values (ML ≥ 70%) is indicated above branches. *Pycnora sorophora* was used as outgroup. https://binary.pensoft.net/file/1413677This dataset is made available under the Open Database License (http://opendatacommons.org/licenses/odbl/1.0/). The Open Database License (ODbL) is a license agreement intended to allow users to freely share, modify, and use this Dataset while maintaining this same freedom for others, provided that the original source and author(s) are credited.Kerry Knudsen, Jana Kocourková, Eva Kondrysová, Tereza Pušová, Jason Hollinger, Steve Leavitt, John McCarthy, Lucie Jedličková, Martin Westberg

Supplementary material 3 *Acarospora joshuaensis* (K.Knudsen & Kocourk.) K. Knudsen & Kocourk., comb. nov. Data type pdf https://binary.pensoft.net/file/1413678This dataset is made available under the Open Database License (http://opendatacommons.org/licenses/odbl/1.0/). The Open Database License (ODbL) is a license agreement intended to allow users to freely share, modify, and use this Dataset while maintaining this same freedom for others, provided that the original source and author(s) are credited.Kerry Knudsen, Jana Kocourková, Eva Kondrysová, Tereza Pušová, Jason Hollinger, Steve Leavitt, John McCarthy, Lucie Jedličková, Martin Westberg
