# Supplementary material for: ﻿Discovering the diversity of Acarosporaceae (Acarosporales, Lecanoromycetes) with carbonized epihymenial accretions in North America
Source: MycoKeys. 2025 Sep 11;122:123–48. doi: 10.3897/mycokeys.122.162675 (PMC12447084; doi:10.3897/mycokeys.122.162675)
Supplement: Supplementary material 1 — Sampled specimens included in the molecular phylogeny [file mycokeys-122-123-s001.pdf]

Supplemental Materials No 1. Table of sampled specimens included in the molecular phylogeny.

**Species**	**Origin**	**Voucher**	**ITS**	**mtSSU**	**nLSU**	**β-TUB**
Acarospora anthracina	U.S.A., Utah, Grand County Co., La Sal Range	Leavitt S. 18-629a	PQ146117	PQ149087	PQ186793	PQ280743
Acarospora anthracina	U.S.A., Utah, Washington Co., Big Mts.	Hollinger J. 19709	PQ146131	PQ149102	PQ186806	PQ280744
Acarospora anthracina	U.S.A., Utah, Iron Co., Dixie National Forest	BRY C25453	PQ146128	PQ149099	PQ186803	PQ280745
Acarospora anthracina	U.S.A., Nevada, Lincoln Co., Highland Peak	Hollinger J. 12544	PQ146132	PQ149103	PQ186807	PQ280746
Acarospora anthracina	U.S.A., Nevada, Lincoln Co., Highland Peak	Hollinger J. 12651	PQ146133	PQ149104	PQ186808	-
Acarospora anthracina	U.S.A., Nevada, Lincoln Co., Irish Mts.	Hollinger J. 13181	PQ146134	PQ149105	PQ186809	PQ280747
Acarospora anthracina	U.S.A., Nevada, Lincoln Co., Jumbled Hills	Hollinger J. 3903	PQ146135	-	PQ186810	PQ317749
Acarospora anthracina	U.S.A., Nevada, Lincoln Co., Mormon Mts.	Hollinger J. 23119	PQ146144	PQ149114	PQ186819	PQ317750
Acarospora aquatica	U.S.A., New York	Lendemer 11526	PQ146145	PQ149115	PQ186820	PQ317751
Acarospora atrata	Norway, Vest-Agder	Westberg 08-125 (S F124797)	LN810761	LN810886	LN810761	LN810653
Acarospora atrata	Sweden, Halland	Arup L02737 (LD)	LN810760	LN810885	LN810760	LN810652
Acarospora austrooccidentalis	U.S.A., New Mexico, Oscura	Kocourkova 10842	OQ171068	OQ184780	OQ195833	PQ317752
Acarospora austrooccidentalis	U.S.A., Utah, Kane Co., Glen Canyon NRA	Leavitt S. 19-104v1	PQ146116	PQ149086	PQ186792	PQ399795
Acarospora austrooccidentalis	U.S.A., Utah	Leavitt S. 23067	PQ146120	PQ149091	PQ186797	PQ317753
Acarospora austrooccidentalis	U.S.A., Nevada, Lincoln Co., Jumbled Hills	Hollinger J. 3909	PQ146136	PQ149106	PQ186811	PQ317754
Acarospora austrooccidentalis	U.S.A., Nevada, Lincoln Co., Jumbled Hills	Hollinger J. 3917	PQ146137	PQ149107	PQ186812	PQ317755
Acarospora austrooccidentalis	U.S.A., New Mexico, Oscura	Kocourkova 10842.2	OQ171083	OQ184795	OQ195846	-
Acarospora austrooccidentalis	U.S.A., Oscura	Kocourkova 10902.1	PQ146141	PQ149111	PQ186816	PQ368708
Acarospora austrooccidentalis	U.S.A., Oscura	Kocourkova 10902.2	PQ146142	PQ149112	PQ186817	PQ368709
Acarospora austrooccidentalis	U.S.A., Oscura	Kocourkova 10902.3	PQ146143	PQ149113	PQ186818	PQ368710
Acarospora badiofusca	Sweden, Östergötland	Nordin 5552 (UPS L-124833)	LN810762	LN810887	LN810762	LN810654
Acarospora badiofusca	Sweden, Jämtland	Nordin & Owe-Larsson 36 (UPS)	LN810763	LN810888	LN810763	LN810655
Acarospora brodoana	U.S.A., California, San Bernardino Co.	Knudsen 14712 & Kocourková (S F256014)	LN810882	LN810955	LN810882	-
Acarospora cervina	Switzerland, Valais	Westberg 10-172 (S F177758)	LN810764	LN810889	LN810764	LN810656
Acarospora cervina	Sweden, Uppland	Westberg SAR200 (S)	LN810765	LN810890	LN810765	LN810657
Acarospora destructans	U.S.A., California, Riverside County, Mojave Desert	Kocourkova 10557	OM522311	OM522315	-	PQ368711
Acarospora destructans	U.S.A., Nevada, Lincoln Co., East Mormon Mts.	Hollinger J. 16408	PQ146130	PQ149101	PQ186805	PQ368717
Acarospora destructans	U.S.A., California, Kern Co.	Dart 1490	PQ146129	-	PQ186804	PQ399796
Acarospora fusca	Sweden, Dalarna	Westberg 10-106 (S)	LN810758	LN810883	LN810758	LN810650
Acarospora fusca	Sweden, Dalarna	Westberg 10-108 (S)	LN810759	LN810884	LN810759	LN810651
Acarospora fuscata	Sweden, Gotland	Westberg SAR120 (LD)	LN810766	LN810891	LN810766	LN810658
Acarospora fuscata	Sweden, Hälsingland	Westberg SAR129 (LD)	LN810767	LN810892	LN810767	LN810659
Acarospora glaucocarpa s. str.	Sweden, Gotland	Westberg SAR08 (LD)	LN810768	LN810893	LN810768	LN810660
Acarospora glaucocarpa s. str.	Sweden, Öland	Westberg WE23 (LD)	LN810769	LN810894	LN810769	LN810661
Acarospora heufleriana	Switzerland, Valais	Westberg 10-174 (S F177764)	LN810774	LN810899	LN810774	LN810666
Acarospora hysgina	Sweden, Bohuslän	Westberg & Westberg SAR121 (LD)	LN810783	LN810908	LN810783	LN810675
Acarospora impressula	Norway, Oslo	Westberg 08-107 (S F121708)	LN810776	LN810901	LN810776	LN810668
Acarospora lapponica	Czech Republic, Moravia, Malá Šťáhle	Bouda 923411	OP162380	OP177764	OP216698	PQ204640
Acarospora laqueata	Switzerland, Vallis	Westberg 10-170 (S F177761)	LN810778	LN810903	LN810778	LN810670
Acarospora leavittii	U.S.A., Utah, Wayne Co., Canyolands NP	Hollinger J. 6404	PQ146139	PQ149109	PQ186814	-
Acarospora macrospora	Norway, Oslo	Westberg 08-109 (S F121710)	LN810779	LN810904	LN810779	LN810671
Acarospora macrospora	Sweden, Gotland	Westberg SAR159 (LD)	LN810780	LN810905	LN810780	LN810672

Acarospora minuta	Canada, Newfoundland and Labrador	McCarthy 3349	ON707076	ON715672	ON725167	PQ399794
Acarospora moenium	Sweden, Torne Lappmark	Westberg P116 (S)	LN810782	LN810907	LN810782	LN810674
Acarospora moenium	Sweden, Västmanland	Westberg 09-066 (S F138363)	LN810781	LN810906	LN810781	LN810673
Acarospora murorum	Spain, Andalusia	Westberg SCIN014 (S)	LN810784	LN810909	LN810784	LN810676
Acarospora nevadensis	U.S.A., California, San Bernardino Co.	Knudsen 9408 (S F223070)	LN810804	LN810929	LN810804	LN810696
Acarospora nicolai	U.S.A., Kansas, Ellsworth Co.	Morse 16136 & Logan (S)	LN810785	LN810910	LN810785	LN810677
Acarospora nodulosa	Spain, Andalusia	Westberg SCIN032 (S)	LN810788	LN810913	LN810788	LN810680
Acarospora nodulosa	Spain, Madrid	Westberg 10-215 (S F177732)	LN810789	LN810914	LN810789	LN810681
Acarospora obpallens	U.S.A., California, Orange Co.	Knudsen 9325 (S F256015)	LN810790	LN810915	LN810790	LN810682
Acarospora oligospora	Norway, Oslo	Westberg 08-106 (S F121705)	LN810791	LN810916	LN810791	LN810683
Acarospora oligospora	Sweden, Uppland	Westberg 09-659 & Tibell (S)	LN810792	LN810917	LN810792	LN810684
Acarospora oscurensis	U.S.A., New Mexico, Oscuro	Kocourkova 10795.2	OQ171047	OQ184763	OQ195818	PQ256829
Acarospora oscurensis	U.S.A., New Mexico, Oscuro	Kocourkova 10795.3	OQ171048	OQ184764	OQ195819	PQ256830
Acarospora oscurensis	U.S.A., New Mexico, Oscuro	Kocourkova 10795	OQ171046	OQ184762	OQ195817	PQ368712
Acarospora oscurensis	U.S.A., Nevada, Lincoln Co., Raonbow Canyon	Hollinger J. 23226a	-	PQ149088	PQ186794	PQ368718
Acarospora oscurensis	U.S.A., Utah	Leavitt S. 23028	PQ146118	PQ149089	PQ186795	PQ368719
Acarospora oscurensis	U.S.A., Utah	Leavitt S. 23034	PQ146119	PQ149090	PQ186796	PQ368720
Acarospora oscurensis	U.S.A., Utah	Leavitt S. 23124	PQ146121	PQ149092	PQ186798	-
Acarospora oscurensis	U.S.A., Utah	Leavitt S. 23126	PQ146122	PQ149093	PQ186799	PQ399790
Acarospora oscurensis	U.S.A., Utah	Leavitt S. 23129	PQ146123	PQ149094	-	PQ399791
Acarospora oscurensis	U.S.A., Utah	Leavitt S. 23168	PQ146120	PQ149095	PQ186800	-
Acarospora oscurensis	U.S.A., Utah, Wayne Co.	Hollinger J. 6327	PQ146140	PQ149110	PQ186815	PQ399792
Acarospora oscurensis	U.S.A., New Mexico, Oscuro	Knudsen 19438	OQ171050	OQ184766	OQ195821	PQ256828
Acarospora peltastica	U.S.A., California, Riverside Co.	Knudsen 9505 (S F256017)	LN810805	LN810930	LN810805	LN810697
Acarospora placodiiformis	Spain, Madrid	Westberg 10-211 (S F177733)	LN810795	LN810920	LN810795	LN810687
Acarospora profusa	U.S.A., Utah	Leavitt S. 23187	PQ146125	PQ149096	-	PQ399798
Acarospora profusa	U.S.A., Utah	Leavitt S. 23203	PQ146126	PQ149097	PQ186801	-
Acarospora rosulata	U.S.A., California, Riverside Co.	Knudsen 9509 (S F256011)	LN810796	LN810921	LN810796	LN810688
Acarospora rosulata	Norway, Oppland	Westberg 08-193 (S)	LN810797	LN810922	LN810797	LN810689
Acarospora rugulosa	Norway, Telemark	Westberg 08-119 (S F123671)	LN810798	LN810923	LN810798	LN810690
Acarospora rugulosa	Sweden, Jämtland	Westberg 10-099 (S F177975)	LN810799	LN810924	LN810799	LN810691
Acarospora schleicheri	China, Sichuan	Obermayer 2919 (UPS L-070426)	LN810800	LN810925	LN810800	LN810692
Acarospora schleicheri	U.S.A., Arizona	Sweat & Yansky KGS1196 (UPS L-162697)	LN810801	LN810926	LN810801	LN810693
Acarospora sinopica	Sweden, Bohuslän	Tibell 22676 (UPS L-113079)	DQ374138	DQ374116	LN810870	EU870751
Acarospora sinopica	Sweden, Härjedalen	Wedin 6617 (UPS)	DQ374148	DQ374120	DQ374148	EU870753
Acarospora socialis	U.S.A., California, San Bernardino Co.	Knudsen 9392 (S F256016)	LN810802	LN810927	LN810802	LN810694
Acarospora sp.	Sweden, Gotland	Westberg SAR20 (LD)	LN810803	LN810928	LN810803	LN810695
Acarospora squamulosa	Sweden, Uppland	Westberg 09-222 (S F139588)	LN810793	LN810918	LN810793	LN810685
Acarospora squamulosa	Norway, Sogn og Fjordane	Westberg 08-153 (S)	LN810794	LN810919	LN810794	LN810686
Acarospora wahlenbergii	Sweden, Härjedalen	Westberg SAR91 (LD)	LN810809	LN810934	LN810809	LN810701
Acarospora wahlenbergii	Sweden, Torne Lappmark	Westberg P115 (S)	LN810810	LN810935	LN810810	LN810702
Glypholecia scabra	Norway, Oppland	Westberg 08-232 (S)	LN810811	LN810936	LN810811	LN810703
Myriospora dilatata	Sweden, Lycksele Lappmark	Nordin 5507 (UPS L-124304)	EU870660	EU870712	LN810871	EU870770
Myriospora dilatata	Sweden, Torne Lappmark	Baloch SW116 (S F114109, holotype)	EU870656	EU870708	LN810872	EU870766

Myriospora myochroa	Sweden, Bohuslän	Westberg 06-051 (LD)	EU870677	EU870729	LN810873	EU870788
Myriospora myochroa	Sweden, Härjedalen	Wedin 6617 (UPS)	DQ374148	DQ374120	DQ374148	EU870753
Myriospora rhagadiza	Sweden, Bohuslän	Westberg 06-040 (LD)	EU870647	EU870699	LN810875	EU870757
Myriospora rhagadiza	Sweden, Bohuslän	Westberg 06-034 (LD)	EU870646	EU870698	LN810876	EU870756
Myriospora scabrida	Sweden, Härjedalen	Santesson 33077a (UPS L-529787)	LN810812	LN810937	LN810812	LN810704
Myriospora scabrida	Norway, Troms	Westberg 2824 (LD)	EU870643	EU870695	LN810877	EU870749
Myriospora smaragdula	Sweden, Härjedalen	Ågren 384 (UPS L-098484)	EU870686	EU870738	LN810878	EU870798
Myriospora smaragdula	Sweden, Härjedalen	Wedin 6620 (UPS)	EU870688	EU870740	LN810879	EU870800
Myriospora tangerina	Sweden, Lycksele Lappmark	Wedin 6873 (UPS)	EU870683	EU870735	LN810880	EU870795
Pleopsidium flavum	Austria, Steiermark	Obermayer 7790 (UPS L-105590)	AY853385	AY853336	AY853385	EU870806
Pleopsidium chlorophanum	Sweden, Torne Lappmark	Nordin 4439 (UPS L-076485)	EU870691	EU870743	EU870691	EU870805
Pleopsidium chlorophanum	Sweden, Jämtland	Nordin 6209 (UPS L-179248)	LN810813	LN810938	LN810813	LN810705
Polysporina cyclocarpa	Norway, Soer-Troendelag	Westberg 08-265 (S F123674)	LN810816	LN810941	LN810816	-
Polysporina simplex	Sweden, Dalsland	Westberg 09-455 (S F152846)	LN810820	LN810945	LN810820	LN810711
Polysporina simplex	Sweden, Dalsland	Westberg SAR199 (S)	LN810821	LN810946	LN810821	LN810712
Polysporina simplex	Norway, Oppland	Westberg 08-270 (S F122563)	LN810823	LN810948	LN810823	LN810713
Polysporina simplex	Norway, Oppland	Westberg 08-247 (S F122590)	LN810824	LN810949	LN810824	LN810714
Polysporina simplex	Sweden, Torne Lappmark	Westberg P118 (S)	LN810825	LN810950	LN810825	LN810715
Polysporina simplex	Austria, Salzburg	Westberg SAR273 (S)	LN810826	LN810951	LN810826	LN810716
Polysporina simplex	Norway, Rogaland	Westberg 08-134 (S F123693)	LN810818	LN810943	LN810818	LN810709
Polysporina simplex	Norway, Oppland	Westberg 08-258 (S F122602)	LN810819	LN810944	LN810819	LN810710
Polysporina simplex	Sweden, Bohuslän	Westberg 06-020 (LD 1267752)	LN810827	LN810952	LN810827	LN810717
Polysporina sp.	Spain, Extremadura, Cáceres	Westberg IBE039	ON794212	ON787696	ON964986	PQ399793
Polysporina subfuscescens	Czech Republic, Central Bohemia, Tišice	Kocourkova 10799	PQ226474	PQ238074	-	-
Polysporina subfuscescens	Czech Republic, Central Bohemia, Nižbor	Kocourkova 10803	OQ171054	OQ184770	-	-
Polysporina subfuscescens	Norway, Sogn og Fjordane	Westberg 08-154 (S F152849)	LN810832	LN810958	LN810832	LN810721
Polysporina subfuscescens	Sweden, Skåne	Westberg 09-169 (S F138167)	LN810831	LN810957	LN810831	LN810720
Polysporina subfuscescens	Norway, Rogaland	Westberg 08-136 (S F123694)	LN810833	LN810959	LN810833	LN810722
Polysporina subfuscescens	Norway, Oppland	Westberg 08-240 (S)	LN810836	LN810962	LN810836	LN810725
Polysporina subfuscescens	Sweden, Pite Lappmark	Westberg 09-638 (S)	LN810837	LN810963	LN810837	LN810726
Polysporina subfuscescens	Sweden, Norrbotten	Westberg 09-566 (S)	LN810838	LN810964	LN810838	LN810727
Polysporina subfuscescens	Sweden, Torne Lappmark	Westberg P113 (S)	LN810839	LN810965	LN810839	LN810728
Polysporina subfuscescens	Norway, Sør-Trøndelag	Westberg 12-018 (S)	LN810844	LN810970	LN810844	LN810733
Polysporina subfuscescens	U.S.A., California, San Bernardino Co.	Knudsen 9405 (S)	LN810847	LN810973	LN810847	LN810736
Polysporina subfuscescens	Norway, Oppland	Westberg 08-281 (S F122560)	LN810830	LN810956	LN810830	LN810719
Polysporina subfuscescens	Sweden, Bohuslän	Westberg & Westberg 06-118 (LD 1264167)	LN810848	LN810974	LN810848	LN810737
Pycnora sorophora	Sweden, Härjedalen	Hermansson 7903a (UPS L-111613)	FJ959357	AY853338	AY853387	LN810757
Sarcogyne albothallina	U.S.A., Montana, Chouteasu Co.	Wheeler 3583 (S)	LN810829	LN810954	LN810829	LN810718
Sarcogyne algoviae	Norway, Oppland	Westberg 08-276 (S F122564)	LN810849	LN810975	LN810849	LN810738
Sarcogyne algoviae	Norway, Oppland	Westberg 08-168 (S F122537)	LN810850	LN810976	LN810850	LN810739
Sarcogyne arenosa	U.S.A., California, Los Angeles Co.	Knudsen 11102 & Sagar (S)	LN810851	LN810977	LN810851	LN810740
Sarcogyne clavus	Austria, Steiermark	Obermayer 09129 (GZU 49-2002)	LN810852	LN810978	LN810852	LN810741
Sarcogyne clavus	Sweden, Värmland	Berglund SAR220 (S)	LN810853	-	LN810853	LN810742
Sarcogyne cyclocarpa	Sweden, Torne Lappmark	Westberg P117 (S)	LN810815	LN810940	LN810815	LN810707

Sarcogyne distinguenda	Sweden, Jämtland	Westberg 08-305 (S F120452)	LN810854	LN810979	LN810854	LN810743
Sarcogyne distinguenda	Norway, Hedmark	Haugan H3852 (O L17425)	LN810855	LN810980	LN810855	LN810744
Sarcogyne hypophaea	Sweden, Uppland	Westberg SAR198 (S)	LN810856	LN810981	LN810856	LN810745
Sarcogyne hypophaea	Finland, Varsinais-Suomi	Pykälä 23561 (H)	LN810857	LN810982	LN810857	LN810746
Sarcogyne hypophaeoides	Sweden, Västmanland	Westberg 08-002 (S F119718)	LN810858	LN810983	LN810858	LN810747
Sarcogyne hypophaeoides	Norway, Rogaland	Westberg 08-139 (S F123697)	LN810859	LN810984	LN810859	LN810748
Sarcogyne paradoxa	U.S.A., California, San Bernardino Co.	Knudsen 9409 (S)	LN810814	LN810939	LN810814	LN810706
Sarcogyne urceolata	Slovakia, Presovsky kraj	Westberg s.n. (UPS L-1075710)	PQ249835	PQ249831	PQ249835	PQ399800
Sarcogyne urceolata	Norway, Finnmark	Klepsland s.n. (UPS L-926140)	PQ249836	PQ249832	PQ249836	PQ399799
Sarcogyne urceolata	Norway, Soer-Troendelag	Westberg 08-260 (S F123679)	LN810840	LN810966	LN810816	LN810729
Sarcogyne urceolata	Norway, Hedmark	Westberg 12-011 (S)	LN810842	LN810968	LN810842	LN810731
Sarcogyne urceolata	Sweden, Torne Lappmark	Westberg P119 (S)	LN810843	LN810969	LN810843	LN810732
Taxon No. 1	U.S.A., Utah, Wayne Co., Canyolands NP	Hollinger J. 6321	PQ146138	PQ149108	PQ186813	PQ368714
Taxon No. 2	U.S.A., California, East Camino Cielto at Gibratral Roac	Dart 1218	OQ171121	OQ184832	OQ195881	PQ399797
Taxon No. 3	U.S.A., Utah	Leavitt S. 23354	PQ146127	PQ149098	PQ186802	PQ368715
Taxon No. 4	U.S.A., Utah, Grand County Co., La Sal Range	Leavitt S. 18-603	PQ249837	PQ249833	PQ186791	PQ368713
Taxon No. 4	U.S.A., Idaho, Cuater Co., Sawtooth Mts.	Hollinger 17524	PQ249838	PQ249834	-	PQ368716
Timdalia intricata	Sweden, Torne Lappmark	Westberg P114 (S)	LN810867	LN810992	LN810867	LN810756
Timdalia intricata	Sweden, Härjedalen	Westberg SAR92 (LD)	LN810866	LN810991	LN810866	LN810755
Trimmatothelopsis rhizobola	Sweden, Lule Lappmark	Westberg 2994 (LD)	EU870640	EU870692	LN810868	EU870745
Trimmatothelopsis rhizobola	Sweden, Lule Lappmark	Westberg 3099 (LD)	EU870641	EU870693	LN810869	EU870746
Trimmatothelopsis terricola	U.S.A., California, Los Angeles Co.	Knudsen 11216 & Sagar (S F256012)	LN810806	LN810931	LN810806	LN810698
Trimmatothelopsis terricola	U.S.A., California, Los Angeles Co.	Knudsen 11216 & Sagar (S F256013)	LN810807	LN810932	LN810807	LN810699
