# Supplementary material for: ﻿Discovering the diversity of Acarosporaceae (Acarosporales, Lecanoromycetes) with carbonized epihymenial accretions in North America
Source: MycoKeys. 2025 Sep 11;122:123–48. doi: 10.3897/mycokeys.122.162675 (PMC12447084; doi:10.3897/mycokeys.122.162675)
Supplement: Supplementary material 2 — Maximum likelihood tree obtained by phylogenetic analysis using a combined data set of ITS, mtSSU, nLSU, and β-TUB sequences of 153 members of Acarosporaceae [file mycokeys-122-123-s002.pdf]

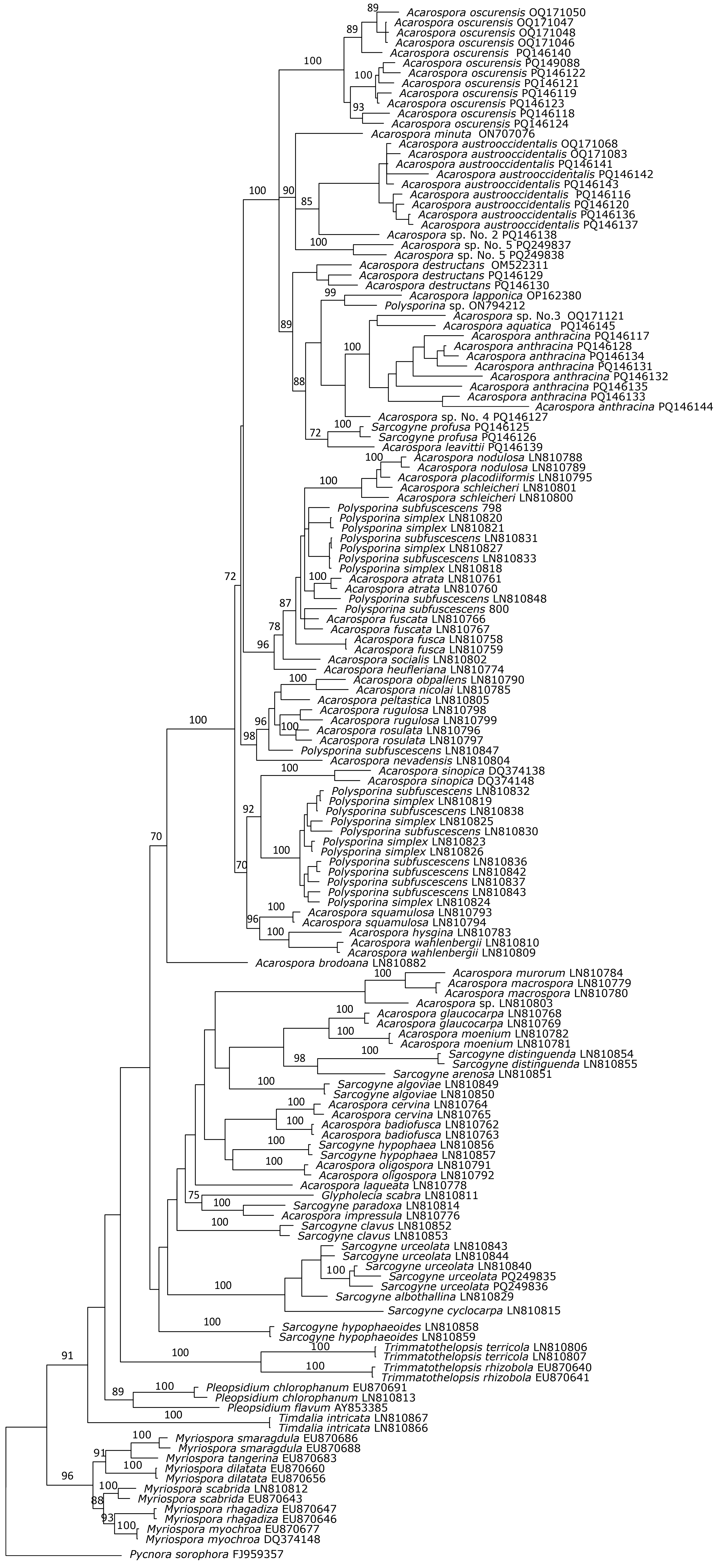

Supplemental Materials No 2 Maximum likelihood tree obtained by phylogenetic analysis using a combined data set of ITS, mtSSU, nLSU, and β-TUB sequences of 153 members of Acarosporaceae. Maximum likelihood bootstrap values (ML ≥ 70%) is indicated above branches. *Pycnora sorophora* was used as outgroup.
