# Supplementary material for: ﻿Discovering the diversity of Acarosporaceae (Acarosporales, Lecanoromycetes) with carbonized epihymenial accretions in North America
Source: MycoKeys. 2025 Sep 11;122:123–48. doi: 10.3897/mycokeys.122.162675 (PMC12447084; doi:10.3897/mycokeys.122.162675)
Supplement: Supplementary material 3 — Acarospora joshuaensis (K.Knudsen & Kocourk.) K. Knudsen & Kocourk., comb. nov. [file mycokeys-122-123-s003.pdf]

Acarospora joshuaensis (K.Knudsen & Kocourk.) K. Knudsen & Kocourk., comb. nov. MB860143

≡*Sarcogyne joshuaensis* K. Knudsen & Kocourk., Bulletin of the California Lichen Society 32(1): 10 (2025).

Type: California, Riverside Co., Joshua Tree National Park, Mojave Desert, upper Juniper Flats, 33.9324, -116.1761, 1479 m, on granite, 18 Dec. 2010, Knudsen 13112.3 (SBBG, holotype), syn. nov.

Description. Squamules, 0.5–2.0(–3.0) mm wide, up to 300 µm thick, with stipe usually half the width of squamules, sometimes wider forming a stout mycelial base, 100–300 µm tall, dispersed or clustered, replicating by division, often becoming contorted, sometimes with lobes. Upper surface usually shiny brown, epruinose. Lower surface white, ecorticate. Epicortex continuous, 10 µm thick. Cortex 40–80 µm thick, of distinct hyphae mostly 2 µm wide, hyaline, usually disarticulated into round cells mostly 2 µm wide. Upper layer cells in brown pigment caps to 4 µm wide and 10 µm thick. Algal layer 50–100 µm thick, algal cells ca.10 µm wide, round to elongate, uninterrupted or occasionally interrupted with distinct narrow bundles of hyphae not forming algal palisades, continuous below apothecia. Medulla up to 100 µm thick of hyphae 2 µm wide, not obscure, merging into the stipe or mycelial base.

Apothecia immersed, disc black, rough, epruinose, punctiform to 1.5 (–2.0 mm) wide. Parathecium indistinct. Hymenium 80–100 µm tal, paraphysis mostly 1.0–1.5 µm wide, apices usually unexpanded in brown gel cap to 3 µm wide, epihymenium 10 µm tall, reddish brown, hymenial gel IKI+ dark blue, subhymenium ca. 20 µm tall, IKI+ dark blue. Asci usually cylindrical 70–90 × 10 (–12) µm, ascospores small mostly 2.0–3.0 × 1.5–2.0 µm. Hypothecium narrow, ca. 10 µm tall, IKI- but often obscured by the IKI+ dark blue of the hymenial gel. Pycnidia ostiole not conspicuous, conidiogenous cells 10–15 × 1 µm, pycnidia mostly 1.0 × 0.5 µm. Not producing secondary metabolites.

Ecology and distribution. Currently known only from three collections in the Mojave Desert in Joshua Tree National Park in southern California on granite from elevations of 1449–1675 m.

Differentiation. *Acarospora joshuaensis* with its large shiny brown convoluted or lobed squamules and black rough immersed apothecia is conspicuous. But it is sympatric with *Sarcogyne fasciculata* on Eureka Peak which is similar). In our key for Acarosporaceae of the southwestern North America (Knudsen et al. 2024), it would be inserted in Section 8, thallus white or brown, not yellow, and key to couplet 9 hymenial gel euamyloid with *Sarcogyne nogalensis* from which it differs in having an indistinct parathecium and squamules instead of areoles.

Specimens examined. California, Riverside Co., Joshua Tree National Park, Mojave Desert, Little San Bernardino Mountains, Eureka Peak, E and west of summit, 34.0325 -116.350278, 1675m, on granite, 18 Dec. 2006, Knudsen et al. 5213 (SBBG), Sheep's Pass, base of Queen Mountain, 34.003111 -116.119972, 1363m, on granite, Dec. 18, 2012, K. Knudsen 13140 (SBBG), upper Juniper Flats, 33.9324, -116.1761, 1479 m, on granite, Dec. 18 2010, Knudsen 13112.2 (SBBG, topotype).

Discussion. Due to an accident, this species was published as *Sarcogyne joshuaensis* but was discussed as *Acarospora joshuaensis.* A new combination is made here. Original publication of article *Acarospora joshuaensis* (Acarosporaceae, Lecenormycetes), a new desert lichen from Southern California in Bulletin of the California Lichen Society 32(1): 9–11 (2025) is open access at https://www.californialichens.org/calsbulleti.

.

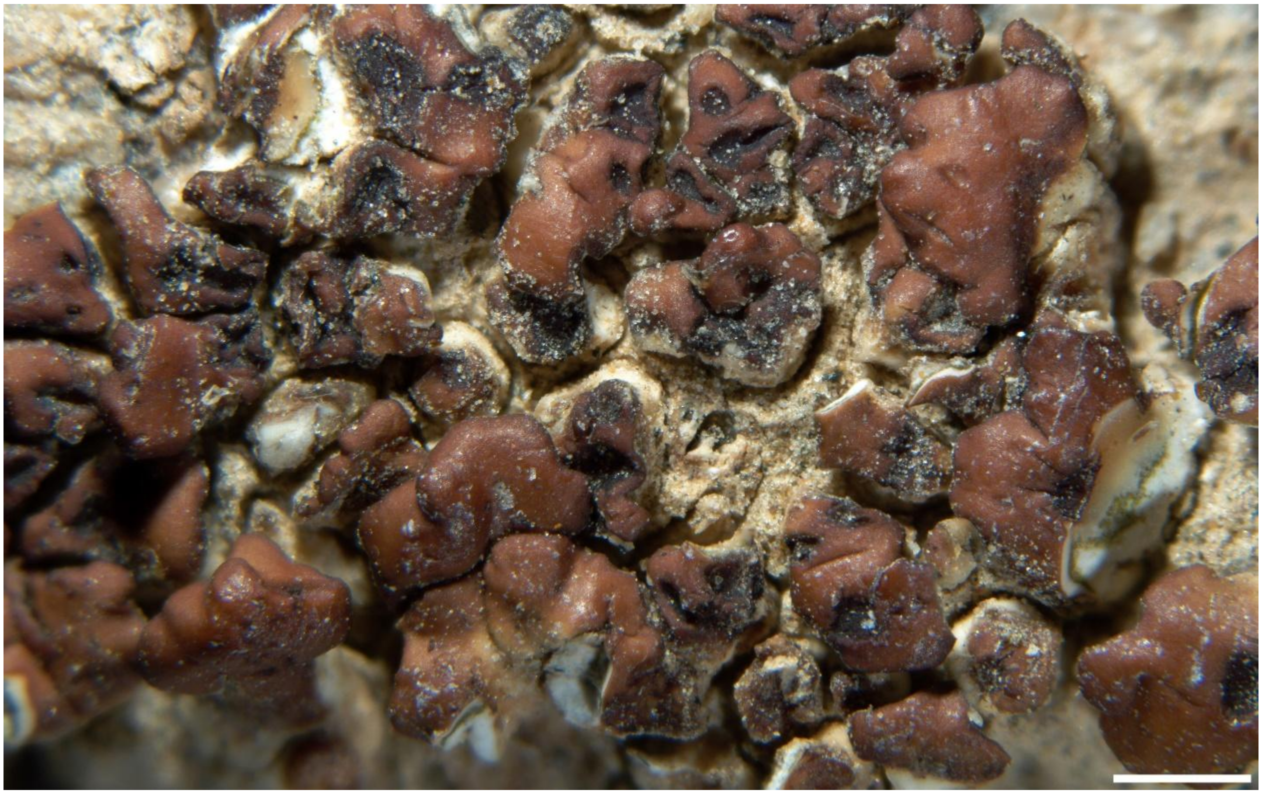

Acarospora joshuaensis, holotype. Bar=1 mm
